# Sexually Dimorphic Vasopressin Cells Modulate Social Investigation and Communication in Sex-Specific Ways

**DOI:** 10.1523/ENEURO.0415-18.2019

**Published:** 2019-01-28

**Authors:** Nicole Rigney, Jack Whylings, Michihiro Mieda, Geert J. de Vries, Aras Petrulis

**Affiliations:** 1 Georgia State University, Atlanta, GA 30303,; 2 Kanazawa University, Kanazawa, Ishikawa, Japan 920-1192

**Keywords:** bed nucleus of the stria terminalis, mice, sex differences, social behavior, social communication, vasopressin

## Abstract

The neuropeptide arginine vasopressin (AVP) has long been implicated in the regulation of social behavior and communication, but precisely which AVP cell groups are involved is largely unknown. To address whether the sexually dimorphic AVP cell group in the bed nucleus of the stria terminalis (BNST) is important for social communication, we deleted BNST AVP cells by viral delivery of a Cre-dependent caspase-3 cell-death construct in AVP-iCre-positive mice using AVP-iCre negative littermate as controls, and assessed social, sexual, aggressive and anxiety-related behaviors. In males, lesioning BNST AVP cells reduced social investigation of other males and increased urine marking (UM) in the presence of a live female, without altering ultrasonic vocalizations (USVs), resident-intruder aggression, copulatory behavior, anxiety, or investigation of females or their odor cues. In females, which have significantly fewer AVP cells in the BNST, these injections influenced copulatory behavior but otherwise had minimal effects on social behavior and communication, indicating that these cells contribute to sex differences in social behavioral function.

## Significance Statement

Despite over thirty years of indirect evidence implicating sexually-dimorphic arginine vasopressin (AVP) cells within the bed nucleus of the stria terminalis (BNST) in the control of social and anxiety-related behavior in mammals, the function of these cells has never been directly tested. Here, we show that deletion of these cells in male, but not female, mice reduce investigation of same-sex conspecifics and alter social communication without changing aggressive and anxiety-related behaviors. Although AVP cell deletion in the BNST did not alter male copulatory behavior, it impaired female sexual behavior. These results indicate that sexually-dimorphic AVP cells in the BNST drive specific aspects of sexually-differentiated social investigation, behavior, and communication.

## Introduction

Social behavior and communication show profound sex differences in many species (Darwin, 1871; Bradbury and Vehrencamp, 1998), and in humans, dysfunction in social behavior and communication is prominent in chronic, debilitating, and pervasive psychopathologies (Insel, 2010) that show sex differences in prevalence and clinical outcome (Halladay et al., 2015), such as autism (Schultz, 2005). One reasonable hypothesis is that sex differences in the underlying neural circuitry contribute to sexually differentiated function and dysfunction in social behavior and communication. A particularly well-positioned circuit in this respect is the arginine vasopressin (AVP) innervation of the brain, which shows marked sex differences across many species, including humans (Goodson and Bass, 2001; De Vries and Panzica, 2006; de Vries, 2008). Research across taxa confirms an important role for AVP in social behavior. For example, AVP has been implicated in aggression, pair bonding, maternal behavior, and communication across vertebrates (Goodson and Bass, 2001; Albers, 2012). However, the anatomy of AVP projections suggests that AVP control of social behavior is complex and, in most cases, the anatomic substrate of AVP’s control of social behavior is unclear (Kelly and Goodson, 2013a; Ludwig and Stern, 2015; Dumais and Veenema, 2016). In most animals, AVP is synthesized in several cell groups, each of which projecting to distinct brain areas (De Vries and Boyle, 1998; Rood and De Vries, 2011; Rood et al., 2013). AVP cells in the medial amygdala (MeA) and bed nucleus of the stria terminalis (BNST) contribute to the most pronounced sex differences in AVP innervation in brain (De Vries and Boyle, 1998). For example, male rats and mice have about two to three times as many AVP cells as females in these nuclei and the projections of these cells to areas such as lateral septum (LS) are denser as well (van Leeuwen et al., 1985; Rood and De Vries, 2011; Rood et al., 2013; Otero-Garcia et al., 2014).

Various studies suggest that AVP cells in the BNST and MeA modulate pro-social as well as antagonistic behaviors. In birds, for example, partial knock-down of AVP gene expression in the BNST reduces prosocial vocalizations and social interactions in birds while increasing male-male aggression (Kelly et al., 2011; Kelly and Goodson, 2013a,b). Evidence for involvement of AVP projections from the BNST and MeA in social behavior in mammals is less direct. For example, the density of AVP fibers in BNST and MeA projection areas and c-Fos activation in AVP cells in the BNST correlate negatively with aggression in male mice and rats (Compaan et al., 1993; Everts et al., 1997; Ebner et al., 2000; Beiderbeck et al., 2007; Veenema et al., 2010) but positively with prosocial behavior (Goodson et al., 2009; Ho et al., 2010). In addition, injecting specific V1a receptor agonists or boosting V1a receptor expression in target areas of AVP cells in the BNST and MeA promotes affiliation in voles (Wang et al., 1994; Liu et al., 2001; Pitkow et al., 2001; Lim and Young, 2004; Lim et al., 2004) and social recognition and active social behaviors in rats (Dantzer et al., 1988; Veenema et al., 2012). In rats, where AVP release in the septum, one of the most prominent projection areas of BNST and MeA AVP cells (De Vries and Buijs, 1983; De Vries and Panzica, 2006), correlates positively with intermale aggression in a resident-intruder test, a behavioral response could be blocked by retro-dialysis of an AVP antagonist (Veenema et al., 2010). None of these results, however, can be tied with certainty to AVP cells in the BNST and MeA, as all these areas receive AVP input from other sources as well, most importantly the PVN (Rood et al., 2013). In addition, AVP released dendritically from neurosecretory neurons in the hypothalamus may reach these areas as well (Ludwig and Stern, 2015). To directly test the hypothesis that AVP cells in the BNST modulate social behavior, we injected an adeno-associated virus (AAV) with a Cre-dependent, genetically modified executioner caspase-3 complex (Yang et al., 2013; Unger et al., 2015) into the BNST of adult AVP-iCre+ and AVP-iCre– (Mieda et al., 2015) male and female mice, which specifically deleted AVP cells in the area, and tested the effects of these deletions on social investigation, courtship ultrasonic vocalizations (USVs; Chabout et al., 2015), and territorial urine marking (UM; Arakawa et al., 2008b), all aspects of mouse communication known to show pronounced sex differences (Crawley, 2012; Lehmann et al., 2013; Wöhr, 2014).

## Materials and Methods

### Animals and husbandry

All mice were maintained at 22°C on a 12/12 h reverse light/dark cycle with food and water available ad libitum, housed in individually ventilated cages (Animal Care Systems), and provided with corncob bedding, a nestlet square, and a housing tube. All animal procedures were performed in accordance with the Georgia State University animal care committee regulations and the National Institutes of Health Guide for the Care and Use of Laboratory Animals.

#### Subjects

Founding AVP-iCre mice were obtained from Dr. Michihiro Mieda (Kanazawa University, Japan). These mice were generated using a bacterial artificial chromosome (BAC) that expressed codon-improved Cre recombinase (Shimshek et al., 2002) under the transcriptional control of the AVP promoter (AVP-iCre mice). In these animals, iCre expression is found in the BNST and the MeA, as well as in hypothalamic areas (Mieda et al., 2015). Subjects were derived by crossing heterozygous iCre+ mutants to wildtype C57Bl/6J mice and genotyped (ear punch) by polymerase chain reaction (PCR) at 21–24 d of age (Transnetyx). Both iCre+ and iCre– littermates were used in behavioral experiments. All subject mice were singly-housed for a minimum of one week.

#### Stimulus animals

CD1(ICR) (Charles River Laboratories) mice were used as stimuli for behavioral testing and to provide male and female subjects with social experience because strain differences between subjects and stimulus mice increase social investigation (Gheusi et al., 1994). Mice were used at 9–16 weeks of age and were novel and unrelated to the subject to which they were exposed.

Female stimulus mice were group-housed, ovariectomized, and implanted with an estradiol capsule (GDX+E), and given two sexual experiences before testing. Two groups of stimulus males were used for behavioral testing. Mice that were used as subordinate mice in the home cage aggression tests and for providing aggressive experience to subjects, were group housed, gonadectomized (GDX), and subjected to two aggressive encounters with a dominant male. Mice in the second group, which provided sexual experience to female subjects and served as sexual partners during copulatory tests and as stimulus animal in the three-chamber social test, were singly-housed, GDX, and implanted with testosterone (GDX+T), and given two sexual experiences before testing.

### Viral vector

**Figure 1.**
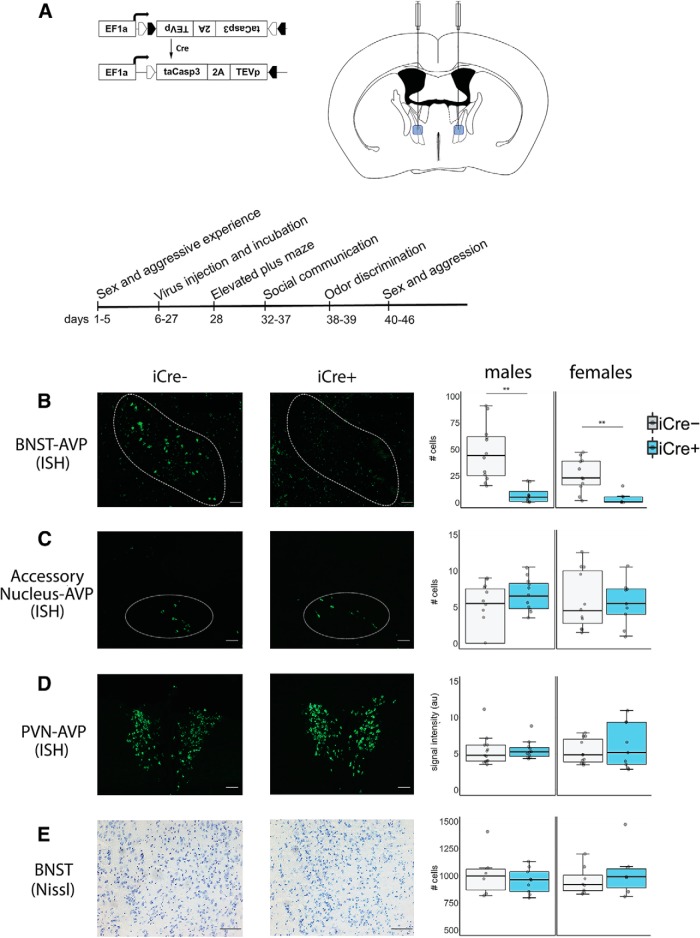
AVP histology and experiment timeline. ***A***, Cre-dependent AAV (AAV-flex-taCasp3-TEVp) and location of bilateral BNST injection site; coordinates: AP –0.01 mm; ML ±0.75 mm; DV 4.8 mm; modified from Paxinos and Franklin (2012). Timeline of experimental manipulations. ***B***, Example images of fluorescent *in situ* hybridization (ISH)-labeled BNST AVP cells and boxplot of cell number. Within the BNST, a significant decrease in AVP cell label was observed in both iCre+ male and female mice compared to iCre– control animals (males: *p* = 0.00014; females: *p* = 0.0025). iCre– (*n* = 13) and iCre+ (*n* = 11) males and iCre– (*n* = 13) and iCre+ (*n* = 8) females. ***C***, Example images of fluorescent ISH-labeled accessory nucleus-AVP cells and boxplot of cell number. No significant AVP cell loss was observed between iCre+ and iCre– subjects (males: *p* = 0.98; females: *p* = 0.89). iCre– (*n* = 13) and iCre+ (*n* = 11) males and iCre– (*n* = 13) and iCre+ (*n* = 8) females. ***D***, Example images of fluorescent ISH-labeled PVN and boxplot of image intensity (arbitrary units). iCre+ and iCre– subjects did not differ in PVN signal intensity (males: *t*_(20)_ = 0.66, *p* = 0.947; females: *p* = 0.29). iCre– (*n* = 13) and iCre+ (*n* = 10) males and iCre– (*n* = 13) and iCre+ (*n* = 8) females. ***E***, Example images of Nissl-stained BNST tissue and boxplot of cell number. No difference in BNST cell number between iCre+ and iCre– subjects was observed (males: *p* = 0.439; females: *p* = 0.44). iCre– (*n* = 6) and iCre+ (*n* = 9) males and iCre– (*n* = 8) and iCre+ (*n* = 6) females. In boxplots, dots indicate individual data points, bold horizontal lines illustrate the median, the areas above and below the lines show the 1st/3rd quartile. The vertical bars range from the minimal to the maximal values excluding outliers (±1.35 SDs from interquartile range). Images were taken at 10× for fluorescent material and 20× for Nissl-stained tissue. Scale bar = 50 µm; ** indicates significant effect of genotype, *p* < 0.005.

AVP driven-, Cre-expressing-BNST neurons were ablated using an AAV (AAV-flex-taCasp3-TEVp; serotype 2/1; 3 × 10^12^ IU/ml; University of North Carolina at Chapel Hill Vector Core) that encodes, in a Cre-dependent fashion, a mutated pro-caspase-3 and its activator (TEVp; Fig. 1*A*). This system activates an apoptotic signaling cascade, cleaving multiple structural and regulatory proteins critical for cell survival and maintenance (Yang et al., 2013) and thereby inducing far less inflammation than other lesion approaches (Morgan et al., 2014).

### Surgery

All surgeries were conducted using 1.5–3% isoflurane gas anesthesia in 100% oxygen; 3 mg/kg of carprofen was given before surgery to reduce pain.

#### Stereotaxic surgery

Mice were positioned in a stereotaxic frame (David Kopf Instruments) with ear and incisor bars holding bregma and lambda level. After a midline scalp incision, a hand operated drill was used to make holes in the skull exposing the dura. For all subjects, 500 nl of AAV-flex-taCasp3-TEVp was delivered bilaterally to the BNST (coordinates: AP –0.01 mm; ML ±0.75 mm; DV 4.8 mm; Paxinos and Franklin, 2012) at a rate of 100 nl/min using a 5-μl Hamilton syringe with a 30-gauge beveled needle mounted on a stereotaxic injector. Following virus delivery, the syringe was left in place for 15 min and slowly withdrawn from the brain.

#### Gonadectomy and hormone treatment

Testes were cauterized and removed at the ductus deferens via a midline abdominal incision. SILASTIC capsules (1.5-cm active length; 1.02-mm inner diameter, 2.16-mm outer diameter; Dow Corning Corporation) were filled with crystalline T (Sigma) and inserted subcutaneously between the scapulae after gonadectomy; this procedure leads to physiologic levels of T (Barkley and Goldman, 1977; Matochik et al., 1994). To further reduce aggression in stimulus animals (Beeman, 1947), some males were GDX, but did not receive a T implant (GDX).

The ovaries of stimulus female mice were removed by cauterization at the uterine horn and attendant blood vessels. SILASTIC capsules (0.7-cm active length; 1.02-mm inner diameter, 2.16-mm outer diameter; Dow Corning Corporation) containing estradiol benzoate (E; diluted 1:1 with cholesterol) were implanted subcutaneously in the scapular region immediately following ovariectomy (GDX+E; Bakker et al., 2002; Ström et al., 2012). To induce sexual receptivity, stimulus females were injected subcutaneously with 0.1 ml of progesterone (500 μg dissolved in sesame oil, Sigma) 4 h preceding sexual experience, urine collection, and behavioral testing (Veyrac et al., 2011).

### Social experience

As opposite-sex sexual experience and attaining competitive status (“social dominance”) promote male and female communicative behaviors (Lumley et al., 1999; Roullet et al., 2011), mice received social experience over five consecutive days (sexual encounters on days 1 and 4, aggressive encounters on days 2 and 5, and no encounters on day 3).

#### Sexual experience

Subjects were given two opportunities to interact with either a stimulus female (for male subjects) or a stimulus male (for female subjects). A sexually-experienced stimulus mouse was placed in the subject’s home cage and removed 5 min after one ejaculation or 90 min in the absence of ejaculation. Subjects that did not show ejaculation (two iCre– males) or did not elicit ejaculation (one iCre+ female) on either trial were removed from further testing.

#### Aggressive experience

Male subjects were exposed to two interactions with subordinate males treated with 40 μl of GDX+T male urine applied to their backs. Gonadectomy, group housing, and social defeat of our subordinates reduce offensive aggression in mice, while GDX+T male urine provides subjects with a male urinary cue that elicits offensive aggression (Beeman, 1947; Connor and Winston, 1972; Van Loo et al., 2001). Subordinate stimulus males were placed in the subject’s home cage and removed after the subject’s first offensive attack (biting) within a 10-min period. All subject males attacked the intruder male stimulus by the second encounter, and all subordinate stimulus males displayed submissive behavior, defined as defensive postures (e.g., on-back), fleeing, and non-social exploring (Koolhaas et al., 2013). Female subjects were exposed to a female intruder; however, this did not elicit any attacks from either animal.

### Experimental procedure

All testing occurred within the first 6 h of the dark cycle under red light illumination, with the exception of the elevated plus maze (EPM). All tests were scored by an experimenter blind to the genotype of the subject. Three to four weeks after viral injections, subjects were habituated to the testing room and apparatus by handling and placing mice (for 5 min) in the three-chamber apparatus (see below) each day for 3 d. On experimental days, subjects were adapted to the experimental room for 15 min before testing. First, we tested mice on an EPM to test for anxiety-related behavior (Lister, 1987). Mice were then tested in the three-chamber apparatus over six consecutive days with a day off on the fourth day. Lastly, odor discrimination, copulatory, and aggressive behavior were measured in the subject’s home cage (Fig. 1*A*). Female subjects were tested irrespective of estrous cycle day, except during copulation testing, when they were in behavioral estrus. Prior research indicates minimal effects of estrous cycle on female mouse communicative behavior (Maggio and Whitney, 1985; Coquelin, 1992; Moncho-Bogani et al., 2004). Following testing, subjects were killed and their brain tissue was processed for *in situ* hybridization to detect AVP expression in BNST and nearby hypothalamic areas.

### Social behavior

USV, UM, and social investigation were recorded in an acrylic three-chamber apparatus (Crawley, 2007; Arakawa et al., 2008a; Moy et al., 2009; Harvard Apparatus; dimensions: 20.3 × 42 × 22 cm). Instead of a solid floor, the apparatus was placed on absorbent paper (Nalgene Versi-dry paper, Thermo Fisher Scientific) so as to accurately measure UM. Animals were also tracked using motion detection software (ANY-maze, San Diego Instruments, RRID:SCR_014289). During testing with stimulus animals, subjects had access to either a stimulus animal in a cage [8 cm (D), 18 cm (H); 3-mm diameter steel bars, 7.4 mm spacing] or an empty cage placed at opposite corners of the outermost chambers of the apparatus. For testing with social odors, subjects had access to 50 μl of fresh urine from a stimulus animal or 50-μl saline pipetted onto a clean piece of filter paper (3 cm^2^), that was taped on the outside of cages. The location of stimulus and the “clean” cage were counterbalanced across animals. After placing the subject in the center of the middle chamber, we measured, across a 5-min trial, close investigation of clean and stimulus cages, distance traveled throughout the apparatus, time spent in the stimulus and clean cage chambers as well as USVs and UMs. After testing, the apparatus and cages were thoroughly cleaned with 70% ethanol and allowed to dry before further testing. In all cases, urine stimulus from one sex was presented first followed by a live stimulus of that same sex; this order was then repeated for the opposite sex. In this fashion, mice experienced first weak (urine) then stronger social stimuli (stimulus animal; the order of male and female stimuli presentation was counterbalanced.

#### Investigation and USVs

Close investigation was defined as time spent sniffing within 2 cm of the stimulus or clean cage; climbing on the cage was not scored as investigation. USVs were detected using a condenser microphone connected to an amplifier (UltraSoundGate CM16/CMPA, 10–200 kHz, frequency range) placed 4 cm inside the apparatus and directly above the center compartment. USVs were sampled at 200 kHz (16-bit) with target frequency set to 70 kHz (UltraSoundGate 116Hb, Avisoft Bioacoustics). Recordings were then analyzed using a MATLAB (MathWorks, RRID:SCR_001622) plug-in that automates USV analysis (Van Segbroeck et al., 2017). Using this program, sonograms were generated by calculating the power spectrum on Hamming windowed data and then transformed into compact acoustic feature representations (Gammatone Filterbank). Each 200-ms window containing the maximum USV syllable duration was then clustered, via machine learning algorithms, into USV syllable types (repertoire units) based on time-frequency USV shape. Repertoire units that appeared as background noise were discarded. We counted the number of all USV produced by each subject. USV syllable types were identified from a subset of males (iCre– *n* = 6; iCre+ *n* = 7) using criterion previously described: short, composite, downward, upward, 1 frequency jump, modulated, multiple frequency jumps, u-shape, flat, chevron (Hanson and Hurley, 2012).

#### UM

Following testing, the substrate sheet was allowed to dry for 1 h and then sprayed with ninhydrin fixative (LC-NIN-16; Tritech Forensics Inc.) to visualize urine marks (Meyer, 1957; Lehmann et al., 2013). After 24 h, sheets were imaged (Sony DSC-S700 camera), binarized and analyzed using a computer-aided imaging software (ImageJ, RRID:SCR_003070). UM was measured as the total area (cm^2^) of visualized ninhydrin urine marks in the entire arena. Urine marks that were larger than 6 cm^2^ and directed toward corners were counted as eliminative “pools” and were counted separately (Bishop and Chevins, 1987).

#### Copulatory and aggressive behavior

To measure copulatory behavior, the stimulus mouse was placed in the subject’s home cage and then removed 5 min after one ejaculation had occurred or if 90 min had elapsed without copulation. The latency and total time investigating the anogenital region, latency to mount, percentage of females that were mounted, percentage of male ejaculations, and number of mount rejections (female kicking male off during mounting attempt) in female subjects was recorded. To measure territorial aggression, subordinate stimulus males were placed in the subject’s home cage and then removed after the subject’s first offensive attack (biting) within a 10-min period; the latency to first bite was recorded.

### Odor discrimination

We used a habituation-discrimination procedure on a subset of subjects to test whether they could distinguish between social odors (Baum and Keverne, 2002). Subjects were given five consecutive 2-min presentations of an odor stimulus with one min intervals between presentations. Subjects were first presented with deionized water, followed by two non-social odors (100% almond, lemon, or vanilla extract, Frontier Natural Products), and then two urine samples (one from each sex) within their home cage. The sequence of odor presentation was counterbalanced within non-social or social odor category. Each odor stimulus (30 μl) was placed onto a clean piece of filter paper (3 cm^2^) taped to an empty food hopper such that subjects could contact the urine samples. Time spent sniffing within 2 cm of the filter paper was recorded. Food hoppers were cleaned with 70% ethanol and allowed to dry between each odor presentation.

### EPM

The EPM consisted of two open arms (30 × 5 cm) and two closed arms (30 × 25 × 5 cm) crossed perpendicularly and raised 60 cm above the floor. Subjects were placed at the arm intersection facing the open arm and were allowed to habituate to the apparatus for 1 min; subjects were then observed for an additional 5 min. Animals were tracked by ANY-maze so that measurements of time spent in open and closed arms were recorded automatically whereas the number of risk assessment behaviors (stretch-attend posture, head-dips) were manually scored from video (Cole and Rodgers, 1993).

### Urine collection

Pooled urine samples were collected from stimulus females induced into estrus and from stimulus males (five to eight mice per sample). Estrous state was verified by color, swelling, and expanded size of vaginal opening (Caligioni, 2009). To collect urine, mice were picked up by the tail base and held by dorsal neck skin; this method was often sufficient to induce urination. If the mouse did not urinate, stroking its belly from an anterior to posterior direction stimulated bladder voiding. Each mouse provided 15–50 μl of urine that was pooled into a 1.5-ml Eppendorf tube. Urine samples were used fresh within 1 h of collection to prevent chemosignal degradation (Roullet et al., 2011).

### Histology and *in situ* hybridization

Following testing, subjects were killed via CO_2_ asphyxiation. Brains were extracted and flash frozen via submersion in 2-methyl-2-butanol (Sigma) for 10–20 s and stored at −80°C until sectioned. Coronal sections (20 μm) were cut with a cryostat (Leica CM3050 S, Leica Biosystems) into three series and stored at −80°C. All tissue was handled in a RNase-free environment. Tissue was postfixed in paraformaldehyde, followed by a wash in 2× saline-sodium citrate (SSC) and acetylation in a triethanolamine/acetic anhydride solution, rinsed in dH_2_O, washed in acetone/methanol solutions (1:1), and again in 2× SSC. Tissue was first incubated at 65°C in hybridization buffer (50% deionized formamide, 1% yeast tRNA, 10% dextran sulphate, 1× Denhardt’s solution, 5% 20× SSC) for 30 min before probe application. Riboprobes were developed from linearized PK Bluescript SK(+) with inserted mouse-vasopressin gene (NM_027106.4, Genscript) using digoxygenin (DIG)-conjugated uracil. Riboprobe synthesized from this plasmid was added to hybridization buffer at a concentration of 100 ng/100 μl and denatured at 90°C for 5 min. Tissue was then hybridized at 65°C for 24 h in a humid chamber. The tissue was then subjected to two 10-min washes in 2× SSC at room temperature followed by a 15-min digestion with RNase A (10 g/ml in 2× SSC) at 37°C. This was followed by a 30-min 2× SSC wash at 56°C and two 10-min 2× SSC washes at room temperature. The tissue was then quenched in 1% H_2_O_2_ in 1× SSC for 15 min, rinsed twice in 1× SSC with 0.1% Tween followed by one 5-min TBS (20 mM Tris and 150 mM NaCl, pH 7.6) wash. Blocking solution (normal sheep serum and bovine caesin) was applied and tissue was incubated for 30 min followed by a 2-h, room temperature incubation with anti-DIG-HRP (1:200, Roche Applied Sciences). Unbound antibody was washed away with three 10-min washes in TBS-T (0.05% Tween in TBS). DIG-labeled probe signal was amplified and visualized using a TSA Plus Fluorescein kit (PerkinElmer) by incubating sections in a 1:50 dilution of the Fluorescein working solution for 12 min followed by three 10-min washes in TBS. Tissue was then cover-slipped using Prolong Gold (Life Technologies) for subsequent imaging and tissue analysis. Tissue processed using sense RNA probe generated no specific labeling. A subset of brain sections was Nissl stained to determine whether viral vector injections resulted in a non-specific loss of BNST cells.

### Tissue analysis

Bilateral images were taken at 10× magnification using a Zeiss Axio Imager.M2 microscope (Carl Zeiss Microimaging), which transferred fluorescent images (FITC contrast reflector) to image analysis software (Stereo Investigator, MicroBrightField, RRID:SCR_002526). Imaging domains (2 mm^2^) were placed with reference to anatomic landmarks (ventricles, fiber tracts; Paxinos and Franklin, 2012). Fluorescently labeled AVP mRNA-expressing cells were counted in the BNST in both hemispheres and averaged over three sections covering the extent of the AVP cell population in the BNST. In addition, we counted nearby accessory AVP mRNA expressing cells (Rood and De Vries, 2011) as well as average label intensity for AVP mRNA in the paraventricular nucleus of the hypothalamus (PVN; ImageJ) to determine any possible off-target effects of our injections. Although we counted AVP mRNA-expressing cells in the PVN as well, AVP mRNA label intensity was chosen as the preferred method for quantification due to the difficulty of discriminating between overlapping AVP cells in PVN. Lastly, we Nissl stained and imaged BNST tissue at 20× magnification to confirm no significant cell loss.

### Statistical analysis

All data were analyzed and graphed in R (3.4.4; R Core Team, 2017). Histology, social investigation, movement, UM (in male subjects), odor discrimination, and EPM data met the assumptions of parametric statistical tests. Therefore, we analyzed histologic data with *t* tests and data on social investigation, movement (distance traveled, time in chambers containing stimulus and clean cages) and male UM with mixed-model ANOVAs [between-subject factor: genotype (iCre+, iCre–); within-subject factors: sex of stimulus (male, female); preference for stimulus (stimulus, clean) followed by *t* tests assessing genotype effects]. Total distance traveled within the apparatus was analyzed using a mixed-model ANOVA (between-subject factor: genotype; within-subject factor: sex of stimulus) as was time spent in open/closed arms in the EPM test (between-subject factor: genotype; within-subject factor: arm); effects of genotype on additional anxiety behaviors (stretch-attend, head-dips) were analyzed using *t* tests. We determined whether subjects could discriminate between odors by comparing odor investigation on the last trial for one odor and odor investigation on the first trial of the subsequent odor using paired *t* tests. The number of female UMs, USVs, USV syllable type, measures of copulatory behavior, and aggression behavior were not normally distributed and could not be transformed, therefore, we analyzed genotype effects using pairwise Mann–Whitney *U* tests. Differences in proportion of animals engaging in copulatory/aggressive behaviors across genotype was assessed using χ^2^ tests. All *post hoc* pairwise comparisons report Bonferroni-corrected *p* values and Cohen’s *d* for effect size when statistically significant. Results were considered significant if *p* < 0.05. All statistical tests are presented in Table 1.

**Table 1:** Statistical analysis

Figure	Data structure	Type of test	Sample size	Statistical data
1*B*, AVP cell count in BNST	Normal distribution	Independent samples *t* test	Males: AVP-iCre– = 13 AVP-iCre+ = 11Females: AVP-iCre– = 13 AVP-iCre+ = 8	Males: *p* = 0.00014 (two-tailed), *t* = 4.57, df = 22; Cohen’s *d* = 2.64Females: *p* = 0.0025 (two-tailed), *t* = 3.58, df = 19; Cohen’s *d* = 2.02
1*C*, AVP cell count in nearby accessory area	Normal distribution	Independent samples *t* test	Males: AVP-iCre– = 13 AVP-iCre+ = 11Females: AVP-iCre– = 13 AVP-iCre+ = 8	Males: *p* = 0.987 (two-tailed) *t* = –0.16, df = 22Females: *p* = 0.89 (two-tailed) *t* = –0.15, df = 19
1*D*, AVP fluorescent intensity (au) in the PVN and cell count in the PVN	Normal distribution	Independent samples *t* test	Males: AVP-iCre– = 13 AVP-iCre+ = 10Females: AVP-iCre– = 13 AVP-iCre+ = 8	Intensity (au) measurement:Males: *p* = 0.947 (two-tailed), *t* = 0.66, df = 20Females: *p* = 0.289 (two-tailed) *t* = –1.10, df = 19Cell counts:Males: *p* = 0.514 (two-tailed) *t* = 0.66, df = 20Females: *p* = 0.82 (two-tailed) *t* = 0.79, df = 19
1*E*, Nissl cell count in BNST	Normal distribution	Independent samples *t* test	Males: AVP-iCre– = 6 AVP-iCre+ = 9Females: AVP-iCre– = 8 AVP-iCre+ = 6	Males: *p* = 0.439 (two-tailed), *t* = 0.79, df = 13Females: *p* = 0.44 (two-tailed), *t* = –0.80, df = 12
2*A*,*B*, social investigation (live animal condition)	Normal distribution	Mixed model analysis with one between-subject factor (genotype) and two repeated measure [sex of stimulus, location of stimulus (two levels)], followed by independent samples *t* test with Bonferroni correction	Males: AVP-iCre– = 13 AVP-iCre+ = 11Females: AVP-iCre– = 13 AVP-iCre+ = 8	Males: *F*_SEX OF STIMULUS(1,22)_ = 261.34, *p* = 1.0792E–13*F*_GENOTYPE(1,22)_ = 0.62 , *p* = 0.438*F*_GENOTYPE × SEX OF STIMULUS(1,22)_ = 5.89, *p* = 0.024*F*_GENOTYPE × LOCATION OF STIMULUS(1,22)_ = 10.71, *p* = 0.003*F*_SEX OF STIMULUS × LOCATION OF STIMULUS(1,22)_ = 59.35, *p* = 1.0976E-7*F*_GENOTYPE × SEX OF STIMULUS × LOCATION OF STIMULUS(1,22)_ = 11.16, *p* = 0.003Investigation of female stimulus: *p* = 1.0 (two-tailed) *t* = –0.70, df = 22Investigation of male stimulus: *p* = 0.004 (two-tailed) *t* = 3.75, df = 22; Cohen’s *d* = 1.52Investigation of clean stimulus in female condition: *p* = 0.70 (two-tailed) *t* = –1.40, df = 22Investigation of clean stimulus in male condition: *p* = 1.0 (two-tailed) *t* = –0.92, df = 22Females:*F*_SEX OF STIMULUS(1,19)_ = 55.92, *p* = 4.4965E-7*F*_GENOTYPE(1,19)_ = 1.60 , *p* = 0.29*F*_GENOTYPE × SEX OF STIMULUS(1,19)_ = 2.16, *p* = 0.161*F*_GENOTYPE × LOCATION OF STIMULUS(1,19)_ = 11.58, *p* = 0.001*F*_SEX OF STIMULUS × LOCATION OF STIMULUS(1,19)_ = 3.12, *p* = 0.09*F*_GENOTYPE × SEX OF STIMULUS × LOCATION OF STIMULUS(1,19)_ = 0.004, *p* = 0.94Investigation of female stimulus: *p* = 0.33 (two-tailed) *t* = 1.82, df = 19Investigation of male stimulus: *p* = 0.51 (two-tailed) *t* = 0.67, df = 19Investigation of clean stimulus in female condition: *p* = 0.58 (two-tailed) *t* = 0.57, df = 19Investigation of clean stimulus in male condition: *p* = 0.06 (two-tailed) *t* = –2.69, df = 19
2*C*,*D*, social investigation (urine condition)	Normal distribution	Mixed model analysis with one between-subject factor (genotype) and two repeated measure [sex of stimulus, location of stimulus (two levels)]	Males: AVP-iCre– = 13 AVP-iCre+ = 11Females: AVP-iCre– = 13 AVP-iCre+ = 8	Males: *F*_SEX OF STIMULUS(1,22)_ = 117.39, *p* = 2.7526E–10 *F*_GENOTYPE(1,22)_ = 0.07 , *p* = 0.79 *F*_GENOTYPE × SEX OF STIMULUS(1,22)_ = 1.31, *p* = 0.26 *F*_GENOTYPE × LOCATION OF STIMULUS(1,22)_ = 0.05, *p* = 0.003 *F*_SEX OF STIMULUS × LOCATION OF STIMULUS(1,22)_ = 59.35, *p* = 0.82 *F*_GENOTYPE × SEX OF STIMULUS × LOCATION OF STIMULUS(1,22)_ = 0.22, *p* = 0.64 Females: *F*_SEX OF STIMULUS(1,19)_ = 60.33, *p* = 2.5924E-7 *F*_GENOTYPE(1,19)_ = 2.9 , *p* = 0.10 *F*_GENOTYPE × SEX OF STIMULUS(1,19)_ = 1.48, *p* = 0.161 *F*_GENOTYPE × LOCATION OF STIMULUS(1,19)_ = 1.16, *p* = 0.30 *F*_SEX OF STIMULUS × LOCATION OF STIMULUS(1,19)_ = 4.27, *p* = 0.53 *F*_GENOTYPE × SEX OF STIMULUS × LOCATION OF STIMULUS(1,19)_ = 2.91, *p* = 0.10

Table 2, social investigation (time spent in zones, live animal condition)	Normal distribution	Mixed model analysis with one between-subject factor (genotype) and two repeated measure [sex of stimulus, location of zone (two levels)]	Males: AVP-iCre– = 13 AVP-iCre+ = 10Females: AVP-iCre– = 13 AVP-iCre+ = 8	Males: *F*_ZONE LOCATION(1,22)_ = 15.68, *p* = 0.001 *F*_GENOTYPE(1,22)_ = 0.0002 , *p* = 0.99*F*_GENOTYPE × SEX OF STIMULUS(1,22)_ = 1.33, *p* = 0.26*F*_GENOTYPE × ZONE LOCATION(1,22)_ = 0.01, *p* = 0.922*F*_SEX OF STIMULUS × ZONE LOCATION(1,22)_ = 14.30, *p* = 0.001*F*_GENOTYPE × SEX OF STIMULUS × ZONE LOCATION(1,22)_ = 0.13, *p* = 0.72Females:*F*_ZONE LOCATION(1,19)_ = 1.56, *p* = 0.23*F*_GENOTYPE(1,19)_ = 1.70 , *p* = 0.21*F*_GENOTYPE × SEX OF STIMULUS(1,19)_ = 2.32, *p* = 0.14*F*_GENOTYPE × ZONE LOCATION(1,19)_ = 2.21, *p* = 0.15*F*_SEX OF STIMULUS × ZONE LOCATION(1,19)_ = 6.73, *p* = 0.017*F*_GENOTYPE × SEX OF STIMULUS × ZONE LOCATION(1,19)_ = 0.04, *p* = 0.85
Table 2, social investigation (distance traveled, live animal condition)	Normal distribution	Mixed model analysis with one between-subject factor (genotype) and one repeated measure (sex of stimulus)	Males: AVP-iCre– = 13 AVP-iCre+ = 10Females: AVP-iCre– = 13 AVP-iCre+ = 8	Males: *F*_SEX OF STIMULUS(1,22)_ = 2.16, *p* = 0.16*F*_GENOTYPE(1,22)_ = 3.48, *p* = 0.33*F*_GENOTYPE × SEX OF STIMULUS(1,22)_ = 0.01, *p* = 0.92Females:*F*_SEX OF STIMULUS(1,19)_ = 0.000043, *p* = 0.96*F*_GENOTYPE(1,19)_ = 3.47, *p* = 0.08*F*_GENOTYPE × SEX OF STIMULUS(1,19)_ = 0.01, *p* = 0.91
Not shown, social investigation (time spent in zones, urine condition)	Normal distribution	Mixed model analysis with one between-subject factor (genotype) and two repeated measure [sex of stimulus, location of zone (two levels)]	Males: AVP-iCre– = 13 AVP-iCre+ = 10Females: AVP-iCre– = 13 AVP-iCre+ = 8	Males: *F*_ZONE LOCATION(1,22)_ = 0.07, *p* = 0.80*F*_GENOTYPE(1,22)_ = 3.21 , *p* = 0.08*F*_GENOTYPE × SEX OF STIMULUS(1,22)_ = 0.52, *p* = 0.48*F*_GENOTYPE × ZONE LOCATION(1,22)_ = 0.75, *p* = 0.40*F*_SEX OF STIMULUS × ZONE LOCATION(1,22)_ = 0.26, *p* = 0.61*F*_GENOTYPE × SEX OF STIMULUS × ZONE LOCATION(1,22)_ = 0.56, *p* = 0.82Females:*F*_ZONE LOCATION(1,19)_ = 1.14, *p* = 0.30 *F*_GENOTYPE(1,19)_ = 4.28 , *p* = 0.54*F*_GENOTYPE × SEX OF STIMULUS(1,19)_ = 0.66, *p* = 0.43*F*_GENOTYPE × ZONE LOCATION(1,19)_ = 5.10, *p* = 0.12*F*_SEX OF STIMULUS × ZONE LOCATION(1,19)_ = 0.19, *p* = 0.67*F*_GENOTYPE × SEX OF STIMULUS × ZONE LOCATION(1,19)_ = 0.0003, *p* = 0.99
Not shown, social investigation (distance traveled, urine condition)	Normal distribution	Mixed model analysis with one between-subject factor (genotype) and one repeated measure (sex of stimulus), followed by independent samples *t* test with Bonferroni correction	Males: AVP-iCre– = 13 AVP-iCre+ = 10Females: AVP-iCre– = 13 AVP-iCre+ = 8	Males: *F*_SEX OF STIMULUS(1,22)_ = 0.73, *p* = 0.79*F*_GENOTYPE(1,22)_ = 2.85, *p* = 0.11*F*_GENOTYPE × SEX OF STIMULUS(1,22)_ = 0.70, *p* = 0.41Females:*F*_SEX OF STIMULUS(1,19)_ = 0.15, *p* = 0.70*F*_GENOTYPE(1,19)_ = 7.6, *p* = 0.012*F*_GENOTYPE × SEX OF STIMULUS(1,19)_ = 0.33, *p* = 0.57distance traveled in male urine condition: *p* = 0.05 (two-tailed), *t* = –2.42, df = 19distance traveled in male urine condition: *p* = 0.19 (two-tailed), *t* = –1.93, df = 19
3*A*,*B*, UM (live animal condition)	Males: normal distribution Females: non-normal	Males: mixed model analysis with one between-subject factor (genotype) and one repeated measure (sex of stimulus), followed by independent samples *t* test with Bonferroni correctionFemales: Mann–Whitney *U* test, independent samples with Bonferroni correction	Males: AVP-iCre– = 13 AVP-iCre+ = 10Females: AVP-iCre– = 13 AVP-iCre+ = 8	Males: *F*_SEX OF STIMULUS(1,22)_ = 52.62, *p* = 0.00000029*F*_GENOTYPE(1,22)_ = 23.72, *p* = .000072*F*_GENOTYPE × SEX OF STIMULUS(1,22)_ = 21.02, *p* = 0.000145UM to female stimulus: *p* = 0.000112 (two-tailed) *t* = –4.6, df = 22; Cohen’s *d* = 2.04UM to male stimulus: *p* = 0.16 (two-tailed) *t* = –1.45, df = 22Females:UM to female stimulus across genotype: *p* = 0.15 (two-tailed), *U* = 20UM to male stimulus across genotype: *p* = 0.32 (two-tailed), *U* = 32
3*C*,*D*, UM (urine condition)	Males: normal distribution Females: non-normal	Males: mixed model analysis with one between-subject factor (genotype) and one repeated measure (sex of stimulus)Females: Mann–Whitney *U* test, independent samples with Bonferroni correction	Males: AVP-iCre– = 13 AVP-iCre+ = 10Females: AVP-iCre– = 13 AVP-iCre+ = 8	Males: *F*_SEX OF STIMULUS(1,22)_ = 0.53, *p* = 0.48*F*_GENOTYPE(1,22)_ = 12.51, *p* = 0.002*F*_GENOTYPE × SEX OF STIMULUS(1,22)_ = 0.16, *p* = 0.70Females:UM to female stimulus across genotype: *p* = 0.467 (two-tailed), *U* = 52UM to male stimulus across genotype: *p* = 0.858 (two-tailed), *U* = 32

4*A*,*B*, USVs (live animal condition)	Non-normal	Mann–Whitney *U* test, independent samples with Bonferroni correction	Males: AVP-iCre– = 13 AVP-iCre+ = 10Females: AVP-iCre– = 13 AVP-iCre+ = 8	Males: USV to female stimulus across genotype: *p* = 1.0 (two sided), *U* = 64USV to male stimulus across genotype: *p* = 0.334 (two sided), *U* = 47Females: USV with male stimulus across genotype: *p* = 0.16 (two-tailed), *U* = 52USV to female stimulus across genotype: *p* = 0.18 (two-tailed), *U* = 58
4*E*,*F*, USVs (live animal condition), syllable type	Non-normal/homogenous	Mann–Whitney *U* test, independent samples with Bonferroni correction	Males: AVP-iCre– = 6 AVP-iCre+ = 7	Males: USV syllable type (all two-tailed):short: *p* = 0.14, *U* = 10, composite: *p* = 1.0, *U* = 22, downward: *p* = 0.63, *U* = 17,upward: *p* = 0.63, *U* = 17, 1 frequency jump: *p* = 0.45, *U* = 15, modulated: *p* = 0.37, *U* = 27, multiple frequency jumps: *p* = 0.63, *U* = 24.5, u-shape: *p* = 0.83, *U* = 19.5, flat: *p* = 0.73, *U* = 18,chevron: *p* = 0.08, *U* = 39
4*C*,*D*, USVs (urine condition)	Non-normal	Mann–Whitney *U* test, independent samples with Bonferroni correction	Males: AVP-iCre– = 13 AVP-iCre+ = 10Females: AVP-iCre– = 13 AVP-iCre+ = 8	Males: USV to female stimulus across genotype: *p* = 0.77 (two sided), *U* = 60USV to male stimulus across genotype: *p* = 0.50 (two sided), *U* = 52Females: USV with male stimulus across genotype: *p* = 0.49 (two sided), *U* = 58USV to female stimulus across genotype: *p* = 0.26 (two sided), *U* = 60
5*A*,*B*, EPM (open/closed arm measurements)	Normal distribution	Mixed model analysis with one between-subject factor (genotype) and one repeated measure (open/closed arm)	Males: AVP-iCre– = 13 AVP-iCre+ = 10Females: AVP-iCre– = 13 AVP-iCre+ = 8	Males: *F*_OPEN/CLOSED ARM(1,22)_ = 51.74, *p* = 7.826E-7*F*_GENOTYPE(1,22)_ = 0.36 , *p* = 0.52*F*_GENOTYPE × OPEN/CLOSED ARM(1,22)_ = 2.81, *p* = 0.11Females: *F*_OPEN/CLOSED ARM(1,19)_ = 189.41, *p* = 2.477E-11*F*_GENOTYPE(1,19)_ = 0.30 , *p* = 0.60*F*_GENOTYPE × OPEN/CLOSED ARM(1,19)_ = 1.30, *p* = 0.59
5*C*,*D*, EPM (additional anxiety measurements)	Normal distribution	Mixed model analysis with one between-subject factor (genotype) and one repeated measure (stretch attends/head dips)	Males: AVP-iCre– = 13 AVP-iCre+ = 10Females: AVP-iCre– = 13 AVP-iCre+ = 8	Males: *F*_STRETCH ATTENDS/HEAD DIPS(1,22)_ = 262.1, *p* = 1.0479E-13*F*_GENOTYPE(1,22)_ = 0.68 , *p* = .42*F*_GENOTYPE × STRETCH ATTENDS/HEAD DIPS(1,22)_ = 3.90, *p* = 0.16Females: *F*_STRETCH ATTENDS/HEAD DIPS(1,19)_ = 33.82, *p* = 0.000016*F*_GENOTYPE(1,19)_ = 2.03 , *p* = 0.17*F*_GENOTYPE × STRETCH ATTENDS/HEAD DIPS(1,19)_ = 0.80, *p* = 0.382
6*A*,*B*, sex behavior (latency to mount, number of female rejections)	Normal distribution	Independent samples *t* test with Bonferroni correction	Males: AVP-iCre– = 13 AVP-iCre+ = 10Females: AVP-iCre– = 13 AVP-iCre+ = 8	Males: latency to mount: *p* = 0.31 (two-tailed), *t* = 1.04, df = 22Females: latency to be mounted: *p* = 0.03 (two-tailed), *t* = –0.52, df = 19number of rejections: *p* = 0.61 (two-tailed), *t* = –0.52, df = 19
6*C*,*D*, sex behavior (percent mounted)	NA	Pearson’s χ^2^	Males: AVP-iCre– = 13 AVP-iCre+ = 10Females: AVP-iCre– = 13 AVP-iCre+ = 8	Males: Pearson’s χ^2^: *p* = 0.34Females: Pearson’s χ^2^: *p* = 1.2797E-27
7*A*, aggressive behavior (latency)	Non-normal	Mann–Whitney *U* test, independent samples	Males: AVP-iCre– = 13 AVP-iCre+ = 10Females: AVP-iCre– = 13 AVP-iCre+ = 8	Males: latency to attack across genotype: *p* = 0.955 (two sided), *U* = 60Females: NA
7*B*, aggressive behavior (percent attacking)	NA	Pearson’s χ^2^	Males: AVP-iCre– = 13 AVP-iCre+ = 10Females: AVP-iCre– = 13 AVP-iCre+ = 8	Males: Pearson’s χ^2^: *p* = 0.85 (two sided)Females: NA
8*A*,*B*, odor discrimination	Normal distribution	Paired samples *t* test with Bonferroni correction between odors	Males: AVP-iCre– = 11 AVP-iCre+ = 5Females: AVP-iCre– = 5 AVP-iCre+ = 7	Males (all two-tailed, iCre– df = 10, iCre+ df = 4):water (3) vs almond (1): *t* = 5.132, *p* = 0.0004 (iCre–), *t*_(4)_ = 5.261, *p* = 0.006 (iCre+)almond (3) vs coconut (1): *t* = 1.21, *p* = 0.20 (iCre–), *t* = 1.725, *p* = 0.16 (iCre+)coconut (3) vs male urine (1): *t* = 11.41, *p* < 0.00001 (iCre–), *t* = 6.675, *p* = 0.003 (iCre+)male urine (3) vs female urine (1): *t* = 7.936, *p* = 0.00001 (iCre–), *t* = 8.313, *p* = 0.001 (iCre+)Females (all two-tailed, iCre– df = 10, iCre+ df = 4):water (3) vs almond (1): *t* = 1.431, *p* = 0.226 (iCre–), *t* = 1.56, *p* = 0.218 (iCre+)almond (3) vs coconut (1): *t* = 2.250, *p* = 0.09 (iCre–), *t* = 2.10, *p* = 0.10 (iCre+)coconut (3) vs male urine (1): *t* = 6.197, *p* = 0.003 (iCre–), *t* = 7.454, *p* = 0.0003 (iCre+)male urine (3) vs female urine (1): *t* = 7.071, *p* = 0.002 (iCre–), *t* = 5.211, *p* = 0.002 (iCre+)

## Results

### Histology

Injection of a viral vector encoding a Cre-dependent cell-death construct into the BNST highly effectively reduced AVP cell numbers in both iCre+ males and females, which had only 10% of the number found in iCre– subjects (males: *t*_(22)_ = 4.57, *p* = 0.00014, *d* = 2.64; females: *t*_(19)_ = 3.58, *p* = 0.0025, *d* = 2.02; Fig. 1*B*), without significantly reducing the number of nearby accessory AVP mRNA-expressing cells (males: *t*_(22)_ = –0.16, *p* = 0.98; females: *t*_(19)_ = –0.15, *p* = 0.89; Fig. 1*C*) or total level of AVP mRNA label in the PVN (males: *t*_(20)_ = 0.66, *p* = 0.947; females: *t*_(19)_ = –1.10, *p* = 0.29; Fig. 1*D*), suggesting that there were no significant off-target effects. We also observed no difference in the number of AVP mRNA-expressing cells in the PVN between genotype (males: *t*_(20)_ = 0.66, *p* = 0.514; females: *t*_(19)_ = 0.79, *p* = 0.82; Table 1). In addition, Nissl-stained tissue from a subset of subjects revealed no overall cell loss in the BNST (males: *t*_(13)_ = 0.79, *p* = 0.439; females: *t*_(12)_ = –0.80, *p* = 0.44 (Fig. 1*E*). Two iCre+ males and two iCre+ females were removed from the analysis as their AVP cell numbers in the BNST were >3 standard deviations above the mean, which we interpreted as off-target injections. Two additional iCre+ females were removed due to unilateral AVP cell loss in the PVN.

### BNST AVP cell ablations in iCre+ males reduced male-male social investigation

**Figure 2.**
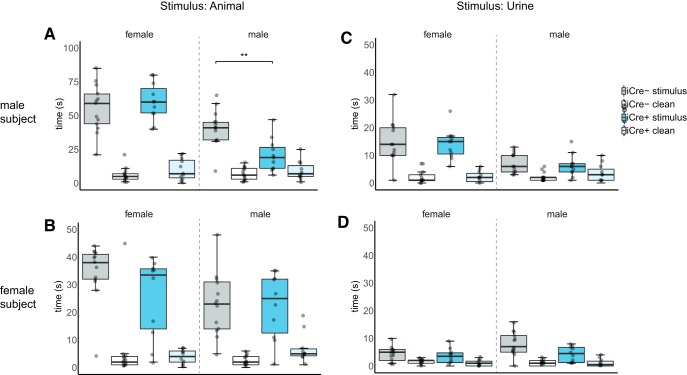
BNST AVP cell ablations in iCre+ males reduced male-male social investigation. Boxplot and individual data points of time spent investigating male or female animals or their urine versus clean control stimuli within the three-chamber apparatus. ***A***, ***B***, Time spent investigating either a caged female versus a clean cage or a caged male versus clean cage. ***A***, iCre– (males: *n* = 13, females: *n* = 13) and iCre+ mice (males: *n* = 11, females: *n* = 8) differed in preference for investigating the stimulus depending on the sex of stimulus (*p* = 0.003). *Post hoc* analysis revealed iCre+ males significantly decreased investigation of the male animal compared to iCre– littermates *p* = 0.004. ***B***, iCre– and iCre+ females did not differ in investigation (*p* = 0.94). ***C***, ***D***, Time spent investigating either female urine or male urine versus saline control placed on filter paper. iCre– and iCre+ subjects did not differ in their investigation of female or male urine. ***C***, Male subjects: *p* = 0.64. ***D***, Female subjects: *p* = 0.10. Note scale difference in animal investigation time between male and female subjects; ** indicates significant effect of genotype, *p* = 0.004. Boxplot representations as in Figure 1.

Mice from both genotypes investigated female stimulus animals more than male stimulus animals (males subjects: *F*_(1,22)_ = 261.34, *p* < 0.00001; female subjects: *F*_(1,19)_ = 55.92, *p* < 0.00001) and had similar overall levels of investigation (male subjects: *F*_(1,22)_ = 0.62, *p* = 0.438; female subjects: *F*_(1,19)_ = 1.60, *p* = 0.29). However, iCre– and iCre+ males differed in preference for investigating the stimulus animal depending on the sex of stimulus (*F*_(1,22)_ = 11.16, *p* = 0.003). *Post hoc* comparisons revealed that iCre+ males significantly decreased investigation of male animals compared to iCre– littermates (*t*_(22)_ = 3.75, *p* = 0.004, *d* = 1.52; Fig. 2*A*) but not female stimulus animals (*t*_(22)_ = –0.70, *p* = 1.0), while ablation of these cells in females did not affect social investigation (*F*_(1,19)_ = 0.004, *p* = 0.94; Fig. 2*B*).

Males of both genotypes investigated female urine more than male urine (*F*_(1,22)_ = 117.39, *p* < 0.00001), whereas female subjects investigated male urine more than female urine (*F*_(1,19)_ = 60.33, *p* < 0.00001). iCre+ mice did not investigate urine differently from iCre– littermates (males: *F*_(1,22)_ = 0.22, *p* = 0.64; females: *F*_(1,19)_ = 2.91, *p* = 0.10; Fig. 2*C*,*D*).

### BNST AVP cell ablations in iCre+ animals did not alter the amount or spatial distribution of activity

Overall, mice of both genotypes traveled similar distances throughout the three-chamber apparatus (males: *F*_(1,22)_ = 3.48, *p* = 0.33; females: *F*_(1,19)_ = 3.47, *p* = 0.08); this pattern did not differ when presented with female or male stimulus animals (males: *F*_(1,22)_ = 0.01, *p* = 0.92; females: *F*_(1,19)_ = 0.01, *p* = 0.91). There were no differences between genotypes in the amount of time mice spent in the stimulus and clean chamber zones (males: *F*_(1,22)_ = 0.13, *p* = 0.72; females: *F*_(1,19)_ = 0.04, *p* = 0.85; Table 2).

**Table 2.** Table of median (interquartile range) distance traveled and time spent in stimulus or clean cage chamber

	Male subjects				Female subjects			
iCre–		iCre+		iCre–		iCre+		
Stimulus	Female	Male	Female	Male	Female	Male	Female	Male
Distance traveled (m)	0.33 (0.22–0.54)	0.32 (0.16–0.5)	0.34 (0.19–0.48)	0.29 (0.13–0.44)	0.28 (0.08–0.38)	0.23 (0.01–0.47)	0.35 (0.16–0.42)	0.34 (0.04–0.5)
Time in stimulus chamber (s)	185 (129–248)	127 (78–195)	175 (111–229)	97 (15–209)	174 (102–246)	147 (30–227)	155.75 (23–198)	119 (91–155)
Time in clean chamber (s)	86 (45–122)	127 (73–155)	90 (49–158)	130 (67–231)	90 (43–176)	113 (52–240)	106 (48–170)	136 (74–282)

iCre– and iCre+ mice did not differ in distance traveled, time spent in animal stimulus, or clean stimulus chambers.

### BNST AVP cell ablated iCre+ males increased UM to females

**Figure 3.**
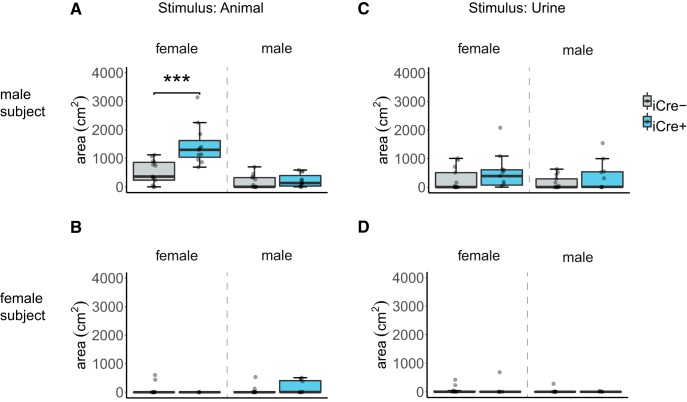
BNST AVP cell ablations in iCre+ males increased UM to females. Boxplot and individual data points of UM in presence of males or females or their urine within the three-chamber apparatus. ***A***, iCre– (males: *n* = 13, females: *n* = 13) and iCre+ mice (males: *n* = 11, females: *n* = 8) differed in UM depending on the sex of stimulus (*p* = 0.00015). *Post hoc* analysis revealed iCre+ males significantly increased UM to the female stimulus compared to iCre– littermates (*p* = 0.000112). ***B***, iCre– and iCre+ females did not differ in UM to stimulus animals [*p* = 0.32 (males), *p* = 0.15 (females)]. ***C***, ***D***, UM with either female urine or male urine present. iCre– and iCre+ subjects did not differ in UM to female or male urine. ***C***, Male subjects: *p* = 0.70. ***D***, Female subjects: *p* = 0.467 (female stimulus), *p* = 0.858 (male stimulus); *** indicates significant effect of genotype, *p* = 0.00015. Boxplot representations as in Figure 1.

Male mice UM more in the presence of females than males (*F*_(1,22)_ = 52.62, *p* < 0.00001. iCre– and iCre+ males differed in overall UM (*F*_(1,22)_ = 23.72, *p* = 0.00007) and UM depending on the sex of stimulus (*F*_(1,22)_ = 21.02, *p* = 0.00015). *Post hoc* comparisons revealed that iCre+ males significantly increased UM to the female stimulus compared to iCre– littermates (*t*_(22)_ = 4.6, *p* = 0.000112, *d* = 2.04; Fig. 3*A*) but not to the male stimulus (*t*_(22)_ = 1.45, *p* = 0.16). iCre– and iCre+ females did not differ in UM in the presence of males (*U* = 32, *p* = 0.32) or females (*U* = 20, *p* = 0.15; Fig. 3*A*). Neither male nor female subjects differed in UM to urine depending on the sex of stimulus [male subjects: *F*_(1,22)_ = 1.16, *p* = 0.70; female subjects: *U* = 52, *p* = 0.467 (female stimulus), *U* = 32, *p* = 0.858 (male stimulus)]. Only one male (iCre+) pooled urine in the male condition, therefore we did not analyze pooled urine.

### BNST AVP cell ablations in iCre+ animals did not alter USVs

**Figure 4.**
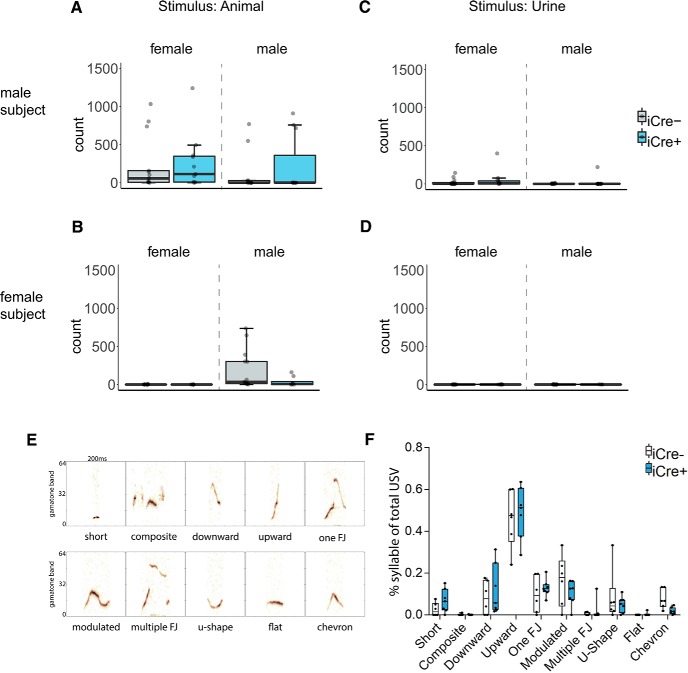
BNST AVP cell ablations in iCre+ animals did not alter USVs. Boxplot and individual data points of USV in presence of a male or female or their urine within the three-chamber apparatus. ***A***, ***B***, iCre– (males: *n* = 13, females: *n* = 13) and iCre+ mice (males: *n* = 11, females: *n* = 8) did not differ by genotype in USV production. ***A***, Male subjects: *p* = 1.0 (female stimulus), *p* = 0.33 (male stimulus). ***B***, Female subjects: *p* = 0.18 (female stimulus), *p* = 0.16 (male stimulus). ***C***, ***D***, USV with either female urine or male urine present. iCre– and iCre+ subjects did not differ in USVs to female or male urine. ***C***, Male subjects: *p* = 0.77 (female stimulus), *p* = 0.5 (male stimulus). ***D***, Female subjects: *p* = 0.26 (female stimulus), *p* = 0.49 (male stimulus). ***E***, USV emitted by male mice were gammatone-transformed (200-ms window) and divided into 10 categories of calls based on spectrographic parameters. ***F***, Male USV syllable type (iCre– *n* = 6; iCre+ *n* = 7). BNST-AVP ablations did not change the percentage of USV syllable types produced between genotypes. Boxplot representations as in Figure 1.

The total number of USVs emitted when iCre– and iCre+ mice were placed with female (male subjects: *U* = 64, *p* = 1.0; female subjects: *U* = 58, *p* = 0.18) or male animals did not differ by genotype (male subjects: *U* = 47, *p* = 0.33; female subjects: *U* = 52, *p* = 0.16; Fig. 4*A*,*B*). Mice from both genotypes also did not differ in USVs to female urine (male subjects: *U* = 60, *p* = 0.77; female subjects: *U* = 53, *p* = 0.26) or male urine (male subjects: *U* = 52, *p* = 0.5; female subjects: *U* = 60, *p* = 0.49; Fig. 4*C*,*D*). Additionally, BNST AVP cell ablations did not change the percentage of USV syllable types produced between male genotypes (short: *U* = 10, *p* = 0.14, composite: *U* = 22, *p* = 1.0, downward: *U* = 17, *p* = 0.63, upward: *U* = 17, *p* = 0.63, 1 frequency jump: *U* = 15, *p* = 0.45, modulated: *U* = 27, *p* = 0.37, multiple frequency jumps: *U* = 24.5, *p* = 0.63, u-shape: *U* = 19.5, *p* = 0.83, flat: *U* = 18, *p* = 0.73, chevron: *U* = 39, *p* = 0.08; Fig. 4*E*,*F*).

### BNST AVP cell ablations in iCre+ animals did not influence anxiety-like behavior

**Figure 5.**
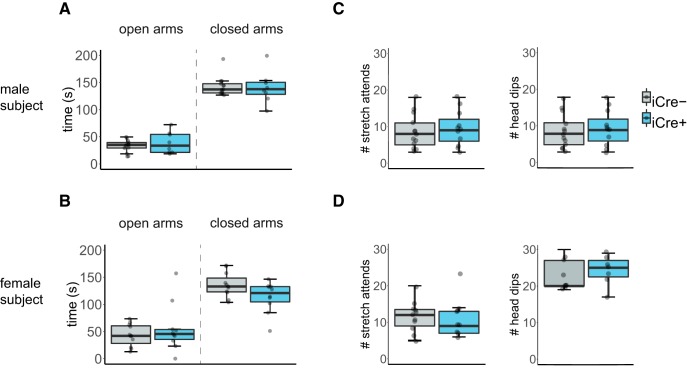
BNST AVP cell ablations in iCre+ animals did not influence anxiety-like behavior. Boxplot and individual data points of time spent in the open and closed arms within the EPM, number of stretch attends, and number of head dips. ***A***, ***B***, iCre– (males: *n* = 13, females: *n* = 13) and iCre+ mice (males: *n* = 11, females: *n* = 8) did not differ by genotype in time spent in open and closed arms. ***A***, Male subjects: *p* = 0.11. ***B***, Female subjects: *p* = 0.59. ***C***, ***D***, iCre– mice (males: *n* = 13, females: *n* = 13) and iCre+ mice (males: *n* = 11, females: *n* = 8) did not differ by genotype in number of stretch attends or head dips. ***C***, Male subjects: *p* = 0.16. ***D***, Female subjects: *p* = 0.38. Boxplot representations as in Figure 1.

All mice spent less time in the open arm than the closed arm of the EPM (male subjects: *F*_(1,22)_ = 51.74, *p* < 0.000001; female subjects: *F*_(1,19)_ = 89.41, *p* < 0.000001). iCre– and iCre+ mice did not differ in the time spent in either arm of the EPM (male subjects: *F*_(1,22)_ = 2.81, *p* = 0.11; female subjects: *F*_(1,19)_ = 1.30, *p* = 0.59; Fig. 5*A*,*B*). Additionally, both genotypes did not differ in frequency of stretch attend postures or head dips (male subjects: *F*_(1,22)_ = 3.90, *p* = 0.16; female subjects: *F*_(1,19)_ = 0.80, *p* = 0.38; Fig. 5*C*,*D*).

### BNST AVP cell ablations in iCre+ animals did not alter male copulatory behavior but did reduce mounting of females

**Figure 6.**
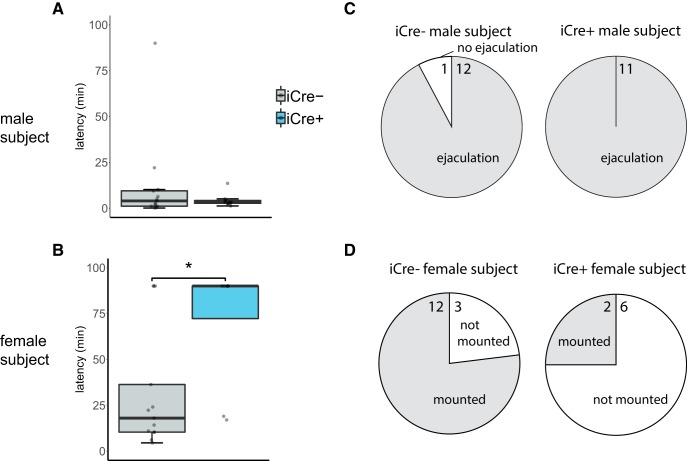
BNST AVP cell ablations in iCre+ animals did not alter male copulatory behavior but did reduce mounting of females. Boxplot and individual data points of male subject’s latency to mount a female (***A***) or female subject’s latency to be mounted (***B***). Pie chart summarizing proportion of male subjects that ejaculated (***C***) or the proportion of female subjects mounted by a male (***D***) with number of subjects in each category indicated. ***A***, ***C***, iCre– (*n* = 13) and iCre+ (*n* = 11) male mice did not differ by genotype in their latency to mount females or in the percentage of subjects ejaculating. ***B***, ***D***, iCre+ (*n* = 8) female mice were mounted at longer latencies (*p* = 0.03) and proportionally less (*p* < 0.000001) than iCre– (*n* = 13) females; * indicates significant effect of genotype, *p* = 0.03. Boxplot representations as in Figure 1.

iCre+ and iCre– males mounted females with similar latencies and did not differ in the percentage of subjects mounting and/or ejaculating (*t*_(22)_ = 1.04, *p* = 0.31; Fig. 6*A*,*C*). However, it took longer for males to mount iCre+ females (*U* = 22, *p* = 0.03) and fewer iCre+ females were mounted overall as compared to iCre– females (χ^2^(2), *p* < 0.000001). However, female iCre+ mice did not reject stimulus males more frequently than did iCre– females (*t*_(19)_ = 0.52, *p* = 0.61). One iCre+ female was removed during the sex behavior test and sex behavior analysis because the stimulus male attacked the female.

### BNST AVP cell ablation did not alter territorial aggression

**Figure 7.**
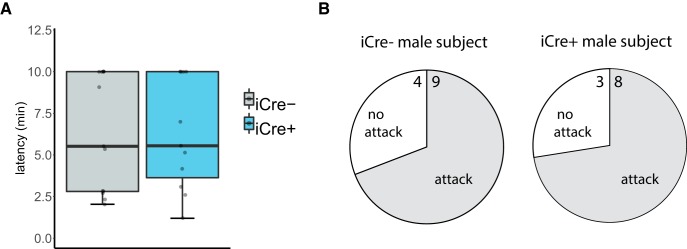
BNST AVP cell ablation did not alter territorial aggression. ***A***, Boxplot and individual data points of male subject’s latency to attack a subordinate intruder male. iCre– (*n* = 13) and iCre+ (*n* = 11) male mice did not differ by genotype in latency to attack the intruder (*p* = 0.955). ***B***, Pie chart summarizing proportion of male subjects that attacked the subordinate intruder in their home cage with number of subjects in each category indicated. Subjects did not differ between genotypes (*p* = 0.85). Boxplot representations as in Figure 1.

The proportion of male subjects that attacked the subordinate intruder in their home cage did not differ between genotypes (χ^2^(2), *p* = 0.85) nor did they differ in attack latency (*U* = 60, *p* = 0.955; Fig. 7*A*,*B*). Female subjects did not attack female intruders.

### BNST AVP cell ablations did not change the ability to discriminate between social odors

**Figure 8.**
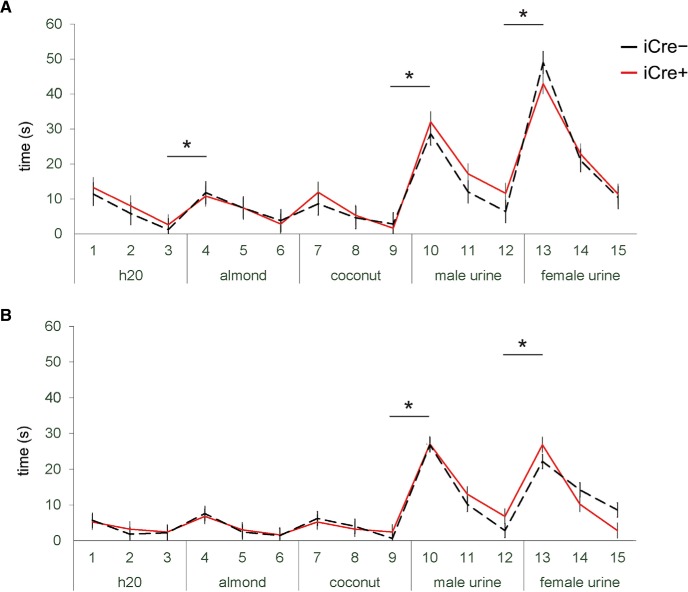
BNST AVP cell ablations did not change the ability to discriminate between social odors. Time spent investigating water, almond or coconut extract, male urine, or female urine. ***A***, Males and ***B***, females of both genotypes were able to discriminate between male and female urine odors [***A***, males: *p* = 0.00001 (iCre−), *p* = 0.001 (iCre+); ***B***, females: *p* = 0.002 (iCre−), *p* = 0.002 (iCre+)] and could distinguish between non-social and social odors [males: *p* < 0.00001 (iCre−), *p* = 0.003 (iCre+); *p* = 0.003 (iCre−), *p* = 0.0003 (iCre+)]. However, subjects’ ability to discriminate between non-social odors was not robust. Although both iCre+ and iCre− males discriminated between water and almond odor, females did not, and no subjects discriminated between the two non-social odors. Data are expressed as mean (±) SEM; trial numbers are given on the x-axis; * indicates significant difference (all *p* < 0.005) between investigation of odors, irrespective of genotype.

Males and females of both genotypes were able to discriminate between male and female urine odors [males: *t*_(10)_ = 7.936, *p* = 0.00001 (iCre–), *t*_(4)_ = 8.313, *p* = 0.001 (iCre+); females: *t*_(4)_ = 7.071, *p* = 0.002 (iCre–), *t*_(6)_ = 5.211, *p* = 0.002 (iCre+)] and could distinguish between non-social and social odors [males: *t*_(10)_ = 11.41, *p* < 0.00001 (iCre–), *t*_(4)_ = 6.675, *p* = 0.003 (iCre+); females: *t*_(4)_ = 6.197, *p* = 0.003 (iCre–), *t*_(6)_ = 7.454, *p* = 0.0003 (iCre+)]. However, subjects’ ability to discriminate between non-social odors was not robust (Fig. 8*A*,*B*). Although both iCre+ and iCre– males discriminated between water and almond odor, females did not, and no subjects discriminated between the two non-social odors (Table 1).

## Discussion

We found that deletion of the sexually dimorphic AVP cell group in the BNST significantly affected social behavior in males, reducing social investigation of other males and increasing UM in the presence of a female. In females, which have significantly fewer AVP BNST cells, similar deletions minimally affected social behavior and communication. This is the first time that direct alteration of a male-biased, sexually-dimorphic population of neuropeptidergic cells has caused a sex difference in mammalian social responses. While several studies have directly tested the social function of sexually-dimorphic cell populations (Yang et al., 2013; Scott et al., 2015; Unger et al., 2015; Wei et al., 2018), only one has found a strong sex difference in social behavior tied to an anatomic sexual dimorphism, in this case a female-biased one (Scott et al., 2015). Our results indicate that sex differences in AVP cells in the BNST contributes to sexually dimorphic components of social communication.

Our experiments do not allow us to conclude that removal of AVP production in the BNST, by and of itself, caused the behavioral effects observed in this study. Lesioning AVP cells also removed all other neuroactive substances co-expressed by these cells, for example, galanin (Miller et al., 1993), which also has been implicated in control of social behavior (Wu et al., 2014). Consequently, our behavioral results may be due to depletion of AVP, or of co-transmitters from BNST projections, or both. Our finding of reduced male-male investigation following BNST AVP cell deletion, however, is strikingly similar to the effects of RNA interference for the homologous peptide arginine vasotocin (AVT) in the BNST of territorial finches (Kelly and Goodson, 2013b) and suggests that this effect is mainly due to a reduction of AVP signal from the BNST.

Our cell deletion approach is, by design, permanent, and so behavioral effects may reflect molecular, cellular, and anatomic adjustments or compensations to chronic depletion of AVP cells in the BNST. Indeed, long-term pharmacological reduction of V1a receptor activity in the LS, a key target of AVP cells in the BNST (De Vries and Panzica, 2006), produces different changes in behavior than acute receptor blockade (Liebsch et al., 1996; Everts and Koolhaas, 1999). A lack of an effect on a behavior, therefore, does not exclude involvement of these cells in that behavior. Nevertheless, our findings of male-specific alterations in social interactions and communication following deletion of AVP cells in the BNST strongly indicate that in males these cells are critical for these functions.

One of the strongest effects of deletion of AVP cells in the BNST was the reduction of same-sex social investigation, which we found in males but not in females. Importantly, this reduction was not due to a lack of general social interest, as investigation of females was unchanged. It was not due to changes in general chemosensory function or motor behavior either, as odor habituation or discrimination of non-social odors and detection of social odors (male or female urine) was unaffected and measures of general activity were also not changed. This reduction can also not be explained by a general increase in anxiety-like behaviors, because the lesions did not affect behavior in the anxiogenic EPM. Instead, it suggests that one function of AVP cells in the BNST is to generate male-typical approach, investigation, and assessment of potential territorial competitors (Oldfield et al., 2015), which is consistent with the observation that knocking down AVT mRNA in the BNST of territorial birds reduces social contact with other males (Kelly and Goodson, 2013b), and that overexpression of V1a receptors (Landgraf et al., 2003) or AVP injections (Koolhaas et al., 1991) in the LS, a key target for AVP projections from the BNST (De Vries and Panzica, 2006), increases active male-male interactions and aggressive behavior in rats. Indeed, retrodialysis of V1aR antagonist into the LS of male rats blocks further territorial aggression during repeated resident-intruder tests (Veenema et al., 2010). Our finding that deleting AVP cells in the BNST reduced investigation of potential competitors is consistent with these findings. Our failure to see a change in aggressive behavior is not. One explanation for this apparent discrepancy is that AVP from other sources contribute to stimulating effects on aggressive behavior in areas such as the LS. For example, AVP cells in the MeA show similar dimorphisms as BNST cells and project to overlapping areas (De Vries and Panzica, 2006). Lesioning both cell groups may be necessary to identify all behaviors in which these sexually dimorphic cell groups are critically involved. Nevertheless, AVP cells in the BNST appear to be required for the proactive investigation of credible social threats rather than for offensive aggression per se.

Ablation of AVP cells in the BNST of male, but not female, mice strongly increased UM toward females, suggesting that these cells normally suppress male scent marking toward females. This increase in UM is not due to excessive eliminative urination because it was not observed toward male stimuli nor was it manifested as an increase in pooled urine. It is possible that this increase is simply due to increased proximity and interest in females. However, males with deletions of AVP cells in the BNST did not differ from control animals in time spent investigating female stimuli, making this explanation less likely. Although UM strongly depends on chemosensory processing (Maruniak et al., 1986; Labov and Wysocki, 1989), the increase in UM by lesioned males was not driven primarily by female urine cues because marking to female urine was not increased, suggesting that other cues, chemosensory as well as non-chemosensory, may drive this increase in scent marking. Given the metabolic costs of urination (Gosling et al., 2000) and the increased risks associated with attraction of aggressive competitors and predators toward urine marks (Desjardins et al., 1973; Roberts et al., 2001), UM should be strongly regulated so that signaling occurs in only specific social contexts. The present results indicate that the BNST-AVP cells may be a key part of the neural system that adaptively regulates this critical signaling behavior.

Although AVP/AVT has been implicated in the production of vocalizations across taxa (Goodson and Bass, 2001), deleting the AVP cells in the BNST did not significantly alter the production of USVs in a reproductive context. This is consistent with the few studies examining male courtship vocalizations (in birds) that report no effect of central infusions of AVT or V1a antagonists on female-directed singing (Goodson and Adkins-Regan, 1999; Goodson and Evans, 2004) or got mixed results from antisense knock-down of BNST-AVP, with one study finding no effect (Kelly et al., 2011) and another observing decreased singing (Kelly and Goodson, 2013b). Most other studies supporting a role for AVP/AVT in vocalizations have not identified the relevant neuroanatomical source of the peptide or its locus of action (Wilczynski et al., 2017) or tended to focus on stress-related or aggressive vocalizations across development (Winslow and Insel, 1993; Bleickardt et al., 2009; Iijima et al., 2014; Lukas and Wöhr, 2015; Paul et al., 2016; Freeman et al., 2018).

Our study also points to a limited, sexually dimorphic role of BNST AVP cells in reproductive behavior. Removal of these cells in males did not alter copulatory behavior, which is consistent with the absence of copulatory deficits in whole-animal knock-outs of V1a or V1b receptors (Wersinger et al., 2004, 2007). However, unexpectedly, ablating these cells in females delayed, and reduced the number of times they were mounted by males. This effect may represent reduced attractiveness of the female or an increased rejection of the male. The latter possibility seems less likely, as we did not detect any change in male investigation toward lesioned females, nor did such females reject males more frequently. It is possible that these females show decreases in cryptic proceptive and/or receptive behaviors. Although AVP has been implicated in female rat sexual behavior, the pattern of prior results is opposite to our findings: in rats, AVP reduced, whereas V1a receptor antagonists increased, female sexual behavior in rats (Södersten et al., 1983; Pedersen and Boccia, 2006). However, as in these studies peptides were injected into the lateral ventricle, it is unclear with what AVP system they interacted.

Although central AVP has been repeatedly implicated in the generation of anxiety states (Ebner et al., 2002; Bielsky et al., 2004; Raggenbass, 2008), we did not observe changes in anxiety-like behavior of lesioned males or females in a non-social anxiogenic environment (Hogg, 1996). This is somewhat surprising because AVP in the LS, an important target of the BNST AVP cells (De Vries and Panzica, 2006), controls anxious states. For example, injections of AVP or V1aR antagonists, or V1aR knock-down within LS all support the idea that AVP is anxiogenic at this site (Landgraf et al., 1995; Liebsch et al., 1996; Beiderbeck et al., 2007). However, since large lesions of BNST have no effect on anxiety-like behavior in the EPM task either (Treit et al., 1998), it may simply mean that, as with aggressive behavior, AVP cells in the BNST are not critically involved in anxiety-like behaviors, and that AVP derived from other sources, such as the PVN or the amygdala (Rood et al., 2013), also drive AVP’s anxiogenic action in the septum and elsewhere.

## Conclusions

A growing body of literature indicates that vasopressin plays a sexually dimorphic role in control of social and anxiety-related behaviors. In humans, for example, intranasal vasopressin stimulates reciprocation of cooperation in males and conciliatory behavior in females, while activating brain areas implicated in reward, social bonding, arousal and memory in males, but not in females (Rilling et al., 2014). In hamsters, hypothalamic injections of vasopressin stimulate aggression in males, while reducing it in females (Terranova et al., 2016). In mice, a genetic knock-out of the vasopressin V1a receptor gene reduces anxiety-related behaviors in males, but not in females (Bielsky et al., 2004, 2005). In rats, a V1a antagonist reduces social play in males while increasing them in females when injected intracerebroventricularly, but has the exact opposite effect when injected into the septum (Veenema et al., 2013). Although, these studies clearly point at a sexually dimorphic role of vasopressin and its cognate receptor in behavior, they do not identify which AVP system is involved, and the opposite effects in play behavior, depending on the area injected, suggests that more than one system may be involved. By targeting a specific AVP cell group directly, our study has identified the sexually dimorphic AVP cells in the BNST as contributing to sex differences in social behavior, and has shown the feasibility of following a similar approach in identifying the contributions of other AVP systems in the brain as well.

## Synthesis

Reviewing Editor: Cheryl Sisk, Michigan State University Decisions are customarily a result of the Reviewing Editor and the peer reviewers coming together and discussing their recommendations until a consensus is reached. When revisions are invited, a fact-based synthesis statement explaining their decision and outlining what is needed to prepare a revision will be listed below. The following reviewer(s) agreed to reveal their identity: Brian Trainor, Matthew Paul.

This manuscript uses a cre-dependent caspase expressing AAV to ablate vasopressin producing neurons in the bed nucleus of the stria terminalis and reports the effects on behavior in male and female mice. The authors use in situ hybridization to validate selective vasopressin knock-down with the bed nucleus and assess behavior across an impressive series of behavioral analyses. Surprisingly, the authors find that the behavioral effects are limited to social approach and scent marking in a sex-dependent fashion: deletion of BNST vasopressin cells leads to a sex-specific actions on social and sexual behaviors: BNST vasopressin deficient males exhibit decreased social investigation of same-sex conspecifics and increased urine marking toward opposite sex conspecifics, whereas BNST vasopressin deficient females exhibit deficits in sex behavior. These results are exciting because the majority of studies assessing the functional effects of vasopressin have relied on pharmacological approaches that target V1a receptors, and so the source of vasopressin is usually unknown. This study introduces a significant advance in the tools used to test the role of individual vasopressin pathways, and represent an important advance in our understanding of population-specific effects of vasopressin neurons.

Both reviewers were enthusiastic about the manuscript and its significance. They offer a number of suggestions for improving the manuscript, primarily by clarifying certain aspects of the methods and results, and by providing additional context and nuance in both the introduction and discussion. Please consider each of the comments below and revise the manuscript accordingly.

Significance Statement

“Despite over thirty years of circumstantial evidence implicating sexually-dimorphic arginine vasopressin (AVP) cells within the bed nucleus of the stria terminalis (BNST) in the control of social and anxiety-related behavior in mammals, the function of these cells has never been directly tested.”

To me, the use of agonists and antagonists at the projection sites of these cells (e.g., lateral septum), is a fairly good test of the function of these AVP cells and is more than circumstantial evidence. What is unique to this study is the ablation of the cell bodies, which impacts AVP function at all target projections - this has never been done before. Rather than referring to the 30 years of research occurring prior to this study as ‘circumstantial,’ the authors could state precisely how their manipulation is different - manipulation of a specific population of AVP cells rather than targets that could receive input from multiple AVP populations. Also, the authors should be careful with their use of the word cells; cells include axons, aka projections.

Introduction

The discussion of the literature on vasopressin role in aggression is oversimplified.

1) The authors claim that, unlike hypothalamic vasopressin, BNST vasopressin appears to regulate prosocial behavior rather than aggression. The authors then go on to point out a negative relationship between vasopressin and aggression. Yet a negative association still suggests regulation of aggression - inhibitory regulation. I believe the authors mean that hypothalamic AVP promotes aggression, whereas BNST AVP promotes prosocial behavior. Instead of using the words ‘mediate’ and ‘regulate,’ the authors may want to consider using ‘stimulate’ or ‘promote.’

2) The authors state that “...release of AVP in the areas that receive BNST projections

correlate negatively with aggression in male mice and rats (Compaan et al., 1993; Everts et al., 1997; Ebner et al., 2000; Beiderbeck et al., 2007; Veenema et al., 2010)...” I believe that Veenema et al. (2010) found a negative correlation of AVP release and aggression in the BNST, but a positive correlation in the lateral septum (a projection of the BNST AVP cells).

Methods and Results

Many parts of the methods section are not written clearly and are difficult to understand. Below are some examples that should be addressed, but the authors may want to consider editing and reorganizing some of the paragraphs/sections to make the methods more readable.

1) I am assuming that the GNX + T stimulus males were used for sex testing with females and GNX - T stimulus males were for male-male aggression tests. If so, it would be helpful to state that explicitly immediately before describing how the gonadectomy and hormone replacement procedures were performed.

2) “...some males were gonadectomized, but did not receive a T implant (GDX + T).”

I am assuming this should be ‘GNX - T’ rather than ‘GNX + T.’

3) Sexual and Aggressive Experience sections.

It would be easier for the reader if the rationales for the sexual and aggressive experience were moved to the first paragraph under the heading, “Social Experience.”

4) “Subordinate stimulus males were placed in the subject's home cage and removed after the subject's first offensive attack (biting) within a ten-minute period. All subject males defeated the intruder male stimulus by the second experience.”

How were the stimulus males defeated if they were removed after the subject's first offensive attack? What were the criteria for ‘defeat?’

5) EPM, “Subjects were placed at the arm intersection facing the open arm and were allowed to explore all arms for one minute; subjects were then observed for five minutes.”

How are subjects observed for 5 minutes if they are only allowed to explore the arms for 1 min?

6) Histology Results: What were the criteria for removing the iCre+ animals? It would seem that an outlier analysis would be the most appropriate method for removal of subjects.

7) All Figure Legends: Much of the text in the results is repeated in the figure legends. Consider removing text in the figure legend to eliminate unnecessary redundancy.

8) Figure 2: a) The legend is confusing. It indicates that the color of the bar graphs represents the Cre genotype of the stimulus, but appears to be the Cre stimulus of the test subject. If true, the clean cage stimuli should also be colored according to the Cre genotype of the test subject. b) Consider increasing the scale on the graphs illustrating female investigation (panels b and d). At the current scale, it is difficult to evaluate the data. It can be noted in the figure legend that the scales of male and female test subject graphs are not equivalent and that they were adjusted to facilitate evaluation of the female test subject data.

9) Table 2: The title of Table 2 should not include statistics. In addition, the authors should check the statistics reported in these two parts of the manuscript as they appear to differ - or perhaps further specification of the precise comparison has been left out?

10) Table 1? There is no Table 1. This is important both for the labeling of Table 2 and the reference to Table 1 in the odor discrimination results section.

11) The authors should state what serotype of AAV was used (AAV2, AAV5, etc).

12) The authors should be consistent with how they report ISH results. Either number of cells or fluorescence intensity for all areas quantified. I also think they should correct for the area quantified (eg cells/mm2).

Discussion

1) “While several studies have directly tested the social function of sexually-dimorphic cell populations (Scott et al. 2015; Unger et al. 2015; Wei et al. 2018; Yang et al. 2013), only one has found a strong sex-difference in social behavior tied to an anatomical sexual dimorphism, in this case a female-biased one (Scott et al. 2015).”

This may be technically true, but manipulations of the sexually dimorphic projections of BNST/MeA vasopressin cells in the septum causes a sex difference in social play behavior (Veenema et al. 2013, Bredewold et al. 2014).

2) Given that vasopressin cells in the medial amygdala are thought to project to the same areas as, and act in concert with, BNST vasopressin cells, it is surprising that this is not mentioned in the discussion. This may be particularly important for the negative results. If vasopressin cells were deleted from both the BNST and medial amygdala the effects may have been even greater or more behaviors may have been affected (e.g., EPM). The authors may want to qualify their conclusions regarding the negative results as medial amygdala vasopressin cells may be compensating for the loss of BNST vasopressin cells.

3) Given the uniqueness of the manipulation in how it impacts all target areas rather than just a single target area, the authors may want to include this in the discussion along with mentioning the putative projection areas of the BNST.

4) The authors state that in species with stronger territoriality, increased AVP/V1a receptor expression is observed in the BNST, then go on to state that their results are inconsistent with these previous findings. I'm not sure I agree with this statement. First, the authors state that the hormone status of intruders in resident-intruder tests may have not provoked aggression. Second, AVP originating in the BNST might be more important for driving social approach to intruders than aggression itself. For example, Duque-Wilckens et al. 2016 reported that inhibition of V1a receptors in the BNST reduced social interaction in males but not offensive aggression.

Minor comments

1) The authors should check the results section for typos, such as this case where the authors include an unnecessary ‘t’ (males: t(22) t = 4.57, p = 0.00014).

2) Histology Results: the text refers to the incorrect panels of Figure 1.

3) Has proceptive behavior (eg ear wiggling etc) ever been quantified in female mice? This could be one way that AVP ablation may have affected the rate at which females were mounted by males.

4) Discussion: “Our experiments do not allow us to conclude that specifically removal of AVP production in the BNST caused the behavioral effects observed in this study.”

Awkward phrasing, consider revising.

## Author Response

Point-by-point response to reviewers' comments

“Both reviewers were enthusiastic about the manuscript and its significance. They offer a number of suggestions for improving the manuscript, primarily by clarifying certain aspects of the methods and results, and by providing additional context and nuance in both the introduction and discussion. Please consider each of the comments below and revise the manuscript accordingly.”

We thank the editor and reviewers for their insightful comments (pasted below in bold), which, we believe, have allowed us to greatly improve the manuscript. Portions of the manuscript that address reviewer's comments are identified by page and line number in our responses and printed in red. We also made a number of grammatical improvements, which we did not highlight.

Significance Statement

“Despite over thirty years of circumstantial evidence implicating sexually-dimorphic arginine vasopressin (AVP) cells within the bed nucleus of the stria terminalis (BNST) in the control of social and anxiety-related behavior in mammals, the function of these cells has never been directly tested.”

To me, the use of agonists and antagonists at the projection sites of these cells (e.g., lateral septum), is a fairly good test of the function of these AVP cells and is more than circumstantial evidence. What is unique to this study is the ablation of the cell bodies, which impacts AVP function at all target projections - this has never been done before. Rather than referring to the 30 years of research occurring prior to this study as ‘circumstantial,’ the authors could state precisely how their manipulation is different - manipulation of a specific population of AVP cells rather than targets that could receive input from multiple AVP populations. Also, the authors should be careful with their use of the word cells; cells include axons, aka projections.

We agree that the agonist and antagonist studies strongly support for a role of AVP projections to the BNST in social behavior in mammals. One of the strongest studies, in this respect was the Veenema et al., 2010 study, mentioned by the reviewer in point #2 for the Introduction. We now highlight that study in the Introduction. We also make clearer that none of these studies can identify with certainty which specific AVP cell systems they targeted, because all of the targeted areas get a mixture of AVP inputs. Rather than using ‘circumstantial,’ which may be taken negatively, we now use ‘indirect.’ We also start with the research in birds, as this will make the distinction between direct and indirect evidence clearer.

‘Introduction’

The discussion of the literature on vasopressin role in aggression is oversimplified.

1) The authors claim that, unlike hypothalamic vasopressin, BNST vasopressin appears to regulate prosocial behavior rather than aggression. The authors then go on to point out a negative relationship between vasopressin and aggression. Yet a negative association still suggests regulation of aggression - inhibitory regulation. I believe the authors mean that hypothalamic AVP promotes aggression, whereas BNST AVP promotes prosocial behavior. Instead of using the words ‘mediate’ and ‘regulate,’ the authors may want to consider using ‘stimulate’ or ‘promote.’

We agree with the reviewer and have changed the text accordingly, now focusing mainly on BNST projections (Page 7, lines 244-252). This version does not use the words ‘mediate’ and ‘regulate’ anymore and indicates the direction of the effect, if appropriate.

“2) The authors state that “...release of AVP in the areas that receive BNST projections correlate negatively with aggression in male mice and rats (Compaan et al., 1993; Everts et al., 1997; Ebner et al., 2000; Beiderbeck et al., 2007; Veenema et al., 2010)...” I believe that Veenema et al. (2010) found a negative correlation of AVP release and aggression in the BNST, but a positive correlation in the lateral septum (a projection of the BNST AVP cells).”

Thank you for catching this. We have corrected this and now give special attention to the Veenema et al., 2010 paper (see our response above) (Page 7, lines 248-264).

Methods and Results

Many parts of the methods section are not written clearly and are difficult to understand. Below are some examples that should be addressed, but the authors may want to consider editing and reorganizing some of the paragraphs/sections to make the methods more readable.

1) I am assuming that the GNX + T stimulus males were used for sex testing with females and GNX - T stimulus males were for male-male aggression tests. If so, it would be helpful to state that explicitly immediately before describing how the gonadectomy and hormone replacement procedures were performed.

We have moved the description of stimulus animals and how they were generated to the section on Stimulus Animals and have deleted redundant text (Page 9, lines 295-302) We have revised this sentence. We also carefully screened the entire Materials and Methods sections and edited sections to make them clearer.

2) “...some males were gonadectomized, but did not receive a T implant (GDX + T).” I am assuming this should be ‘GNX - T’ rather than ‘GNX + T.’

This is now corrected (Page 11, lines 327-328).

3) Sexual and Aggressive Experience sections.

It would be easier for the reader if the rationales for the sexual and aggressive experience were moved to the first paragraph under the heading, “Social Experience.”

We have moved the relevant information under the heading as suggested (Page 11, lines 339-342).

4) “Subordinate stimulus males were placed in the subject's home cage and removed after the subject's first offensive attack (biting) within a ten-minute period. All subject males defeated the intruder male stimulus by the second experience.” How were the stimulus males defeated if they were removed after the subject's first offensive attack? What were the criteria for ‘defeat?’

We have changed the wording of ‘defeat’ to ‘attack’. Stimulus males displayed uniformly defensive behavior (on-back, huddling), while the subjects attacked the stimulus (lunge-bite) (Page 12, lines 355-357).

5) EPM, “Subjects were placed at the arm intersection facing the open arm and were allowed to explore all arms for one minute; subjects were then observed for five minutes.”

How are subjects observed for 5 minutes if they are only allowed to explore the arms for 1 min?

We changed the wording to clarify that the mice were exploring the EPM for 6 minutes total, with a one-minute habituation period before behavior was scored. (Page 15, lines 448-450).

6) Histology Results: What were the criteria for removing the iCre+ animals? It would seem that an outlier analysis would be the most appropriate method for removal of subjects.

iCre+ mice with BNST-vasopressin cell counts higher than 3 standard deviations from the mean of other iCre+ mice were considered ‘misses’ and removed from the study. The criteria of 3+ standard deviations was added to the text. (Page 19, lines 543-545).

7) All Figure Legends: Much of the text in the results is repeated in the figure legends. Consider removing text in the figure legend to eliminate unnecessary redundancy.

We removed redundant statistics that were in the figure legends and left the p values in place for clarity.

8) Figure 2: a) The legend is confusing. It indicates that the color of the bar graphs represents the Cre genotype of the stimulus, but appears to be the Cre stimulus of the test subject. If true, the clean cage stimuli should also be colored according to the Cre genotype of the test subject. b) Consider increasing the scale on the graphs illustrating female investigation (panels b and d). At the current scale, it is difficult to evaluate the data. It can be noted in the figure legend that the scales of male and female test subject graphs are not equivalent and that they were adjusted to facilitate evaluation of the female test subject data.

In figure 2, we have changed the colors for clarity and adjusted the y-axis scale for several graphs to aid in visualizing the data.

9) Table 2: The title of Table 2 should not include statistics. In addition, the authors should check the statistics reported in these two parts of the manuscript as they appear to differ - or perhaps further specification of the precise comparison has been left out?

The statistics are now removed from the title of the table and reported in the text and statistics table (Table 1). We report the F value from the ANOVA, with no post-hoc comparisons because there were no significant effects. The table values represent the median and interquartile range similar to how we visualize our other data in boxplots.

10) Table 1? There is no Table 1. This is important both for the labeling of Table 2 and the reference to Table 1 in the odor discrimination results section.

Table 1 is the statistics table required by the journal and because of its large size. We have placed it at the end of the manuscript for ease of reading.

11) The authors should state what serotype of AAV was used (AAV2, AAV5, etc).

Under the ‘viral vector’ section, we have added additional description of the serotype.

12) The authors should be consistent with how they report ISH results. Either number of cells or fluorescence intensity for all areas quantified. I also think they should correct for the area quantified (eg cells/mm2).

As indicated in the text, the density of AVP cells in PVN made it difficult to accurately count cells in this population. For that reason, we chose to report intensity measurement. However, we now also report approximate cell counts, but doing so does not change outcomes. We have also indicated the size of the counting area (2 mm2). (Page 17-18, lines 498, 504-506, 539-541).

Discussion

1) “While several studies have directly tested the social function of sexually-dimorphic cell populations (Scott et al. 2015; Unger et al. 2015; Wei et al. 2018; Yang et al. 2013), only one has found a strong sex-difference in social behavior tied to an anatomical sexual dimorphism, in this case a female-biased one (Scott et al. 2015).” This may be technically true, but manipulations of the sexually dimorphic projections of BNST/MeA vasopressin cells in the septum causes a sex difference in social play behavior (Veenema et al. 2013, Bredewold et al. 2014).

We agree that these studies provide strong support for a function of the projections from the BNST/MeA in social play, but for reasons we now make clearer (see our response to the comment on the Significance Statement and Introduction above), this study represents the first direct test of the function of the sexually dimorphic AVP cell group in the BNST.

2) Given that vasopressin cells in the medial amygdala are thought to project to the same areas as, and act in concert with, BNST vasopressin cells, it is surprising that this is not mentioned in the discussion. This may be particularly important for the negative results. If vasopressin cells were deleted from both the BNST and medial amygdala the effects may have been even greater or more behaviors may have been affected (e.g., EPM). The authors may want to qualify their conclusions regarding the negative results as medial amygdala vasopressin cells may be compensating for the loss of BNST vasopressin cells.

We actually did mention this in the Discussion, but if the reviewer missed it, so will other readers. Therefore, we now state this potential explanation of negative results more explicitly (Page 29, lines 774-778, and Page 31, last two sentences before ‘Conclusions’).

3) Given the uniqueness of the manipulation in how it impacts all target areas rather than just a single target area, the authors may want to include this in the discussion along with mentioning the putative projection areas of the BNST.

We trust that the changes to the Introduction makes this point clear, and that readers will understand that by deleting the AVP cells in the BNST one automatically deletes the projections of these cells to all target areas as well.

4) The authors state that in species with stronger territoriality, increased AVP/V1a receptor expression is observed in the BNST, then go on to state that their results are inconsistent with these previous findings. I'm not sure I agree with this statement. First, the authors state that the hormone status of intruders in resident-intruder tests may have not provoked aggression. Second, AVP originating in the BNST might be more important for driving social approach to intruders than aggression itself. For example, Duque-Wilckens et al. 2016 reported that inhibition of V1a receptors in the BNST reduced social interaction in males but not offensive aggression.

We agree with the reviewer's concern and now describe previous results more accurately. (Page 28, lines 769-772).

Minor comments

1) The authors should check the results section for typos, such as this case where the authors include an unnecessary ‘t’ (males: t(22) t = 4.57, p = 0.00014).

This is now corrected

2) Histology Results: the text refers to the incorrect panels of Figure 1.

This is now corrected

3) Has proceptive behavior (eg ear wiggling etc) ever been quantified in female mice? This could be one way that AVP ablation may have affected the rate at which females were mounted by males.

Unfortunately, female mice do not display easily quantifiable proceptive behaviors (hop-dart, ear-wiggling in rats). In the future, we will measure female investigation of the male during copulation tests as an additional metric.

4) Discussion: “Our experiments do not allow us to conclude that specifically removal of AVP production in the BNST caused the behavioral effects observed in this study.”

Awkward phrasing, consider revising.

We have revised this sentence. (Page 27, lines 735-736).

## References

[B1] Albers HE (2012) The regulation of social recognition, social communication and aggression: vasopressin in the social behavior neural network. Horm Behav 61:283–292. 10.1016/j.yhbeh.2011.10.007 22079778

[B2] Arakawa H, Arakawa K, Blanchard DC, Blanchard RJ (2008a) A new test paradigm for social recognition evidenced by urinary scent marking behavior in C57BL/6J mice. Behav Brain Res 190:97–104. 10.1016/j.bbr.2008.02.009 18359521 PMC2441767

[B3] Arakawa H, Blanchard DC, Arakawa K, Dunlap C, Blanchard RJ (2008b) Scent marking behavior as an odorant communication in mice. Neurosci Biobehav Rev 32:1236–1248. 10.1016/j.neubiorev.2008.05.012 18565582 PMC2577770

[B4] Bakker J, Honda S-I, Harada N, Balthazart J (2002) The aromatase knock-out mouse provides new evidence that estradiol is required during development in the female for the expression of sociosexual behaviors in adulthood. J Neurosci 22:9104–9112. 10.1523/JNEUROSCI.22-20-09104.2002 12388618 PMC6757696

[B5] Barkley MS, Goldman BD (1977) The effects of castration and Silastic implants of testosterone on intermale aggression in the mouse. Horm Behav 9:32–48. 10.1016/0018-506x(77)90048-4 561020

[B6] Baum MJ, Keverne EB (2002) Sex difference in attraction thresholds for volatile odors from male and estrous female mouse urine. Horm Behav 41:213–219. 10.1006/hbeh.2001.1749 11855906

[B7] Beeman EA (1947) The relation of the interval between castration and first encounter to the aggressive behavior of mice. Anat Rec 99:570. 18935368

[B8] Beiderbeck DI, Neumann ID, Veenema AH (2007) Differences in intermale aggression are accompanied by opposite vasopressin release patterns within the septum in rats bred for low and high anxiety. Eur J Neurosci 26:3597–3605. 10.1111/j.1460-9568.2007.05974.x 18052969

[B9] Bielsky IF, Hu SB, Szegda KL, Westphal H, Young LJ (2004) Profound impairment in social recognition and reduction in anxiety-like behavior in vasopressin V1a receptor knockout mice. Neuropsychopharmacology 29:483–493. 10.1038/sj.npp.1300360 14647484

[B10] Bielsky IF, Hu SB, Young LJ (2005) Sexual dimorphism in the vasopressin system: lack of an altered behavioral phenotype in female V1a receptor knockout mice. Behav Brain Res 164:132–136. 10.1016/j.bbr.2005.06.005 16046007

[B11] Bishop MJ, Chevins PF (1987) Urine odours and marking patterns in territorial laboratory mice (*Mus musculus*). Behav Processes 15:233–248. 10.1016/0376-6357(87)90009-X 24925655

[B12] Bleickardt CJ, Mullins DE, Macsweeney CP, Werner BJ, Pond AJ, Guzzi MF, Martin FDC, Varty GB, Hodgson RA (2009) Characterization of the V1a antagonist, JNJ-17308616, in rodent models of anxiety-like behavior. Psychopharmacology 202:711–718. 10.1007/s00213-008-1354-x 18923820

[B13] Bradbury JW, Vehrencamp SL (1998) Principles of animal communication, Ed 2 Sunderland, MA: Sinauer Associates, Oxford University Press.

[B14] Caligioni CS (2009) Assessing reproductive status/stages in mice. Curr Protoc Neurosci Appendix 4:Appendix 4I. 10.1002/0471142301.nsa04is48 19575469 PMC2755182

[B15] Chabout J, Sarkar A, Dunson DB, Jarvis ED (2015) Male mice song syntax depends on social contexts and influences female preferences. Front Behav Neurosci 9:76. 10.3389/fnbeh.2015.00076 25883559 PMC4383150

[B16] Cole JC, Rodgers RJ (1993) An ethological analysis of the effects of chlordiazepoxide and bretazenil (Ro 16-6028) in the murine elevated plus-maze. Behav Pharmacol 4:573–580. 11224226

[B17] Compaan JC, Buijs RM, Pool CW, De Ruiter AJ, Koolhaas JM (1993) Differential lateral septal vasopressin innervation in aggressive and nonaggressive male mice. Brain Res Bull 30:1–6. 10.1016/0361-9230(93)90032-7 8420617

[B18] Connor JL, Winston H (1972) Genetic analysis of conditioned emotional responses in the mouse (*Mus musculus* L.). J Comp Physiol Psychol 81:37–44. 5074306 10.1037/h0033310

[B19] Coquelin A (1992) Urine-marking by female mice throughout their reproductive cycle. Horm Behav 26:255–271. 10.1016/0018-506x(92)90046-x 1612568

[B20] Crawley JN (2007) Mouse behavioral assays relevant to the symptoms of autism. Brain Pathol 17:448–459. 10.1111/j.1750-3639.2007.00096.x 17919130 PMC8095652

[B21] Crawley JN (2012) Translational animal models of autism and neurodevelopmental disorders. Dialogues Clin Neurosci 14:293–305. 23226954 10.31887/DCNS.2012.14.3/jcrawley PMC3513683

[B22] Dantzer R, Koob GF, Bluthé RM, Le Moal M (1988) Septal vasopressin modulates social memory in male rats. Brain Res 457:143–147. 10.1016/0006-8993(88)90066-2 3167559

[B23] Darwin C (1871) The descent of man, and selection in relation to sex. Princeton, NJ: Princeton University Press.

[B24] Desjardins C, Maruniak JA, Bronson FH (1973) Social rank in house mice: differentiation revealed by ultraviolet visualization of urinary marking patterns. Science 182:939–941. 10.1126/science.182.4115.939 4745598

[B25] De Vries GJ (2008) Sex differences in vasopressin and oxytocin innervation of the brain. Prog Brain Res 170:17–27. 10.1016/S0079-6123(08)00402-0 18655868

[B26] De Vries GJ, Buijs RM (1983) The origin of the vasopressinergic and oxytocinergic innervation of the rat brain with special reference to the lateral septum. Brain Res 273:307–317. 10.1016/0006-8993(83)90855-7 6311351

[B27] De Vries GJ, Boyle PA (1998) Double duty for sex differences in the brain. Behav Brain Res 92:205–213. 10.1016/S0166-4328(97)00192-7 9638962

[B28] De Vries GJ, Panzica GC (2006) Sexual differentiation of central vasopressin and vasotocin systems in vertebrates: different mechanisms, similar endpoints. Neuroscience 138:947–955. 10.1016/j.neuroscience.2005.07.050 16310321 PMC1457099

[B29] Dumais KM, Veenema AH (2016) Vasopressin and oxytocin receptor systems in the brain: sex differences and sex-specific regulation of social behavior. Front Neuroendocrinol 40:1–23. 10.1016/j.yfrne.2015.04.003 25951955 PMC4633405

[B30] Ebner K, Wotjak CT, Landgraf R, Engelmann M (2000) A single social defeat experience selectively stimulates the release of oxytocin, but not vasopressin, within the septal brain area of male rats. Brain Res 872:87–92. 10.1016/S0006-8993(00)02464-1 10924679

[B31] Ebner K, Wotjak CT, Landgraf R, Engelmann M (2002) Forced swimming triggers vasopressin release within the amygdala to modulate stress-coping strategies in rats. Eur J Neurosci 15:384–388. 10.1046/j.0953-816x.2001.01869.x 11849304

[B32] Everts HG, Koolhaas JM (1999) Differential modulation of lateral septal vasopressin receptor blockade in spatial learning, social recognition, and anxiety-related behaviors in rats. Behav Brain Res 99:7–16. 10.1016/s0166-4328(98)00004-7 10512567

[B33] Everts HG, De Ruiter AJ, Koolhaas JM (1997) Differential lateral septal vasopressin in wild-type rats: correlation with aggression. Horm Behav 31:136–144. 10.1006/hbeh.1997.1375 9154434

[B34] Freeman AR, Hare JF, Anderson WG, Caldwell HK (2018) Effects of arginine vasopressin on Richardson’s ground squirrel social and vocal behavior. Behav Neurosci 132:34–50. 10.1037/bne0000225 29553774

[B35] Gheusi G, Bluth R-M, Goodall G, Dantzer R (1994) Social and individual recognition in rodents: methodological aspects and neurobiological bases. Behav Processes 33:59–87. 10.1016/0376-6357(94)90060-4 24925240

[B36] Goodson JL, Adkins-Regan E (1999) Effect of intraseptal vasotocin and vasoactive intestinal polypeptide infusions on courtship song and aggression in the male zebra finch (*Taeniopygia guttata*). J Neuroendocrinol 11:19–25. 9918225 10.1046/j.1365-2826.1999.00284.x

[B37] Goodson JL, Bass AH (2001) Social behavior functions and related anatomical characteristics of vasotocin/vasopressin systems in vertebrates. Brain Res Brain Res Rev 35:246–265. 11423156 10.1016/s0165-0173(01)00043-1

[B38] Goodson JL, Evans AK (2004) Neural responses to territorial challenge and nonsocial stress in male song sparrows: segregation, integration, and modulation by a vasopressin V1 antagonist. Horm Behav 46:371–381. 10.1016/j.yhbeh.2004.02.008 15465522

[B39] Goodson JL, Kabelik D, Schrock SE (2009) Dynamic neuromodulation of aggression by vasotocin: influence of social context and social phenotype in territorial songbirds. Biol Lett 5:554–556. 10.1098/rsbl.2009.0316 19493876 PMC2781940

[B40] Gosling LM, Roberts SC, Thornton EA, Andrew MJ (2000) Life history costs of olfactory status signalling in mice. Behav Ecol Sociobiol 48:328–332. 10.1007/s002650000242

[B41] Halladay AK, Bishop S, Constantino JN, Daniels AM, Koenig K, Palmer K, Messinger D, Pelphrey K, Sanders SJ, Singer AT, Taylor JL, Szatmari P (2015) Sex and gender differences in autism spectrum disorder: summarizing evidence gaps and identifying emerging areas of priority. Mol Autism 6:36. 10.1186/s13229-015-0019-y 26075049 PMC4465158

[B42] Hanson JL, Hurley LM (2012) Female presence and estrous state influence mouse ultrasonic courtship vocalizations. PLoS One 7:e40782. 10.1371/journal.pone.0040782 22815817 PMC3399843

[B43] Hogg S (1996) A review of the validity and variability of the elevated plus-maze as an animal model of anxiety. Pharmacol Biochem Behav 54:21–30. 10.1016/0091-3057(95)02126-4 8728535

[B44] Ho JM, Murray JH, Demas GE, Goodson JL (2010) Vasopressin cell groups exhibit strongly divergent responses to copulation and male-male interactions in mice. Horm Behav 58:368–377. 10.1016/j.yhbeh.2010.03.021 20382147 PMC4195792

[B45] Iijima M, Yoshimizu T, Shimazaki T, Tokugawa K, Fukumoto K, Kurosu S, Kuwada T, Sekiguchi Y, Chaki S (2014) Antidepressant and anxiolytic profiles of newly synthesized arginine vasopressin V1B receptor antagonists: TASP0233278 and TASP0390325. Br J Pharmacol 171:3511–3525. 10.1111/bph.12699 24654684 PMC4105937

[B46] Insel TR (2010) The challenge of translation in social neuroscience: a review of oxytocin, vasopressin, and affiliative behavior. Neuron 65:768–779. 10.1016/j.neuron.2010.03.005 20346754 PMC2847497

[B47] Kelly AM, Goodson JL (2013a) Functional significance of a phylogenetically widespread sexual dimorphism in vasotocin/vasopressin production. Horm Behav 64:840–846. 10.1016/j.yhbeh.2013.09.006 24100197

[B48] Kelly AM, Goodson JL (2013b) Behavioral relevance of species-specific vasotocin anatomy in gregarious finches. Front Neurosci 7:242. 10.3389/fnins.2013.00242 24381536 PMC3865460

[B49] Kelly AM, Kingsbury MA, Hoffbuhr K, Schrock SE, Waxman B, Kabelik D, Thompson RR, Goodson JL (2011) Vasotocin neurons and septal V1a-like receptors potently modulate songbird flocking and responses to novelty. Horm Behav 60:12–21. 10.1016/j.yhbeh.2011.01.012 21295577 PMC3106146

[B50] Koolhaas JM, Moor E, Hiemstra Y, Bohus B (1991) The testosterone-dependent vasopressinergic neurons in the medial amygdala and lateral septum: Involvement in social behaviour of male rats. In Vasopressin, (Jard S, Jamison R, eds), pp 213–219. Paris/London: INSERM/John Libbey Eurotext Ltd.

[B51] Koolhaas JM, Coppens CM, de Boer SF, Buwalda B, Meerlo P, Timmermans PJ (2013) The resident-intruder paradigm: a standardized test for aggression, violence and social stress. J Vis Exp (77):e4367. 10.3791/4367 23852258 PMC3731199

[B52] Labov JB, Wysocki CJ (1989) Vomeronasal organ and social factors affect urine marking by male mice. Physiol Behav 45:443–447. 10.1016/0031-9384(89)90153-4 2756033

[B53] Landgraf R, Gerstberger R, Montkowski A, Probst JC, Wotjak CT, Holsboer F, Engelmann M (1995) V1 vasopressin receptor antisense oligodeoxynucleotide into septum reduces vasopressin binding, social discrimination abilities, and anxiety-related behavior in rats. J Neurosci 15:4250–4258. 10.1523/jneurosci.15-06-04250.1995 7790909 PMC6577715

[B54] Landgraf R, Frank E, Aldag JM, Neumann ID, Sharer CA, Ren X, Terwilliger EF, Niwa M, Wigger A, Young LJ (2003) Viral vector-mediated gene transfer of the vole V1a vasopressin receptor in the rat septum: improved social discrimination and active social behaviour. Eur J Neurosci 18:403–411. 10.1046/j.1460-9568.2003.02750.x 12887422

[B55] Lehmann ML, Geddes CE, Lee JL, Herkenham M (2013) Urine scent marking (USM): a novel test for depressive-like behavior and a predictor of stress resiliency in mice. PLoS One 8:e69822. 10.1371/journal.pone.0069822 23875001 PMC3713058

[B56] Liebsch G, Wotjak CT, Landgraf R, Engelmann M (1996) Septal vasopressin modulates anxiety-related behaviour in rats. Neurosci Lett 217:101–104. 10.1016/0304-3940(96)13069-x 8916082

[B57] Lim MM, Young LJ (2004) Vasopressin-dependent neural circuits underlying pair bond formation in the monogamous prairie vole. Neuroscience 125:35–45. 10.1016/j.neuroscience.2003.12.008 15051143

[B58] Lim MM, Hammock EAD, Young LJ (2004) The role of vasopressin in the genetic and neural regulation of monogamy. J Neuroendocrinol 16:325–332. 10.1111/j.0953-8194.2004.01162.x 15089970

[B59] Lister RG (1987) The use of a plus-maze to measure anxiety in the mouse. Psychopharmacology 92:180–185. 3110839 10.1007/BF00177912

[B60] Liu Y, Curtis JT, Wang Z (2001) Vasopressin in the lateral septum regulates pair bond formation in male prairie voles (*Microtus ochrogaster*). Behav Neurosci 115:910–919. 10.1037//0735-7044.115.4.910 11508730

[B61] Ludwig M, Stern J (2015) Multiple signaling modalities mediated by dendritic exocytosis of oxytocin and vasopressin. Philos Trans R Soc Lond B Biol Sci 370:20140182. 10.1098/rstb.2014.0182 26009761 PMC4455751

[B62] Lukas M, Wöhr M (2015) Endogenous vasopressin, innate anxiety, and the emission of pro-social 50-kHz ultrasonic vocalizations during social play behavior in juvenile rats. Psychoneuroendocrinology 56:35–44. 10.1016/j.psyneuen.2015.03.005 25800147

[B63] Lumley LA, Sipos ML, Charles RC, Charles RF, Meyerhoff JL (1999) Social stress effects on territorial marking and ultrasonic vocalizations in mice. Physiol Behav 67:769–775. 10.1016/s0031-9384(99)00131-6 10604849

[B64] Maggio JC, Whitney G (1985) Ultrasonic vocalizing by adult female mice (*Mus musculus*). J Comp Psychol 99:420–436. 10.1037//0735-7036.99.4.420 4075780

[B65] Maruniak JA, Wysocki CJ, Taylor JA (1986) Mediation of male mouse urine marking and aggression by the vomeronasal organ. Physiol Behav 37:655–657. 10.1016/0031-9384(86)90300-8 3749330

[B66] Matochik JA, Sipos ML, Nyby JG, Barfield RJ (1994) Intracranial androgenic activation of male-typical behaviors in house mice: motivation versus performance. Behav Brain Res 60:141–149. 10.1016/0166-4328(94)90141-4 8003244

[B67] Meyer H (1957) The ninhydrin reaction and its analytical applications. Biochem J 67:333–340. 10.1042/bj0670333 13471556 PMC1200157

[B68] Mieda M, Ono D, Hasegawa E, Okamoto H, Honma K-I, Honma S, Sakurai T (2015) Cellular clocks in AVP neurons of the SCN are critical for interneuronal coupling regulating circadian behavior rhythm. Neuron 85:1103–1116. 10.1016/j.neuron.2015.02.005 25741730

[B69] Miller MA, Kolb PE, Raskind MA (1993) Extra-hypothalamic vasopressin neurons coexpress galanin messenger RNA as shown by double in situ hybridization histochemistry. J Comp Neurol 329:378–384. 10.1002/cne.903290308 7681457

[B70] Moncho-Bogani J, Lanuza E, Lorente MJ, Martinez-Garcia F (2004) Attraction to male pheromones and sexual behaviour show different regulatory mechanisms in female mice. Physiol Behav 81:427–434. 10.1016/j.physbeh.2004.01.014 15135014

[B71] Morgan CW, Julien O, Unger EK, Shah NM, Wells JA (2014) Turning on caspases with genetics and small molecules. Methods Enzymol 544:179–213. 10.1016/B978-0-12-417158-9.00008-X 24974291 PMC4249682

[B72] Moy SS, Nadler JJ, Young NB, Nonneman RJ, Grossman AW, Murphy DL, D’Ercole AJ, Crawley JN, Magnuson TR, Lauder JM (2009) Social approach in genetically engineered mouse lines relevant to autism. Genes Brain Behav 8:129–142. 10.1111/j.1601-183X.2008.00452.x 19016890 PMC2659808

[B73] Oldfield RG, Harris RM, Hofmann HA (2015) Integrating resource defence theory with a neural nonapeptide pathway to explain territory-based mating systems. Front Zool 12:S16. 10.1186/1742-9994-12-S1-S16 26813803 PMC4722349

[B74] Otero-Garcia M, Martin-Sanchez A, Fortes-Marco L, Martínez-Ricós J, Agustin-Pavón C, Lanuza E, Martínez-García F (2014) Extending the socio-sexual brain: arginine-vasopressin immunoreactive circuits in the telencephalon of mice. Brain Struct Funct 219:1055–1081. 10.1007/s00429-013-0553-3 23625152

[B75] Paul MJ, Peters NV, Holder MK, Kim AM, Whylings J, Terranova JI, de Vries GJ (2016) Atypical social development in vasopressin-deficient brattleboro rats. eNeuro 3:ENEURO.0150-15.2016. 10.1523/ENEURO.0150-15.2016 27066536 PMC4822146

[B76] Paxinos G, Franklin K (2012) The mouse brain in stereotaxic coordinates , Ed 4 San Diego, CA: Academic Press.

[B77] Pedersen CA, Boccia ML (2006) Vasopressin interactions with oxytocin in the control of female sexual behavior. Neuroscience 139:843–851. 10.1016/j.neuroscience.2006.01.002 16488546

[B78] Pitkow LJ, Sharer CA, Ren X, Insel TR, Terwilliger EF, Young LJ (2001) Facilitation of affiliation and pair-bond formation by vasopressin receptor gene transfer into the ventral forebrain of a monogamous vole. J Neurosci 21:7392–7396. 10.1523/jneurosci.21-18-07392.2001 11549749 PMC6762997

[B109] R Core Team (2013). R: A language and environment for statistical computing. R Foundation for Statistical Computing, Vienna, Austria.

[B79] Raggenbass M (2008) Overview of cellular electrophysiological actions of vasopressin. Eur J Pharmacol 583:243–254. 10.1016/j.ejphar.2007.11.074 18280467

[B80] Rilling JK, Demarco AC, Hackett PD, Chen X, Gautam P, Stair S, Haroon E, Thompson R, Ditzen B, Patel R, Pagnoni G (2014) Sex differences in the neural and behavioral response to intranasal oxytocin and vasopressin during human social interaction. Psychoneuroendocrinology 39:237–248. 10.1016/j.psyneuen.2013.09.022 24157401 PMC3842401

[B81] Roberts SC, Gosling LM, Thornton EA, McClung J (2001) Scent-marking by male mice under the risk of predation. Behav Ecol 12:698–705. 10.1093/beheco/12.6.698

[B82] Rood BD, De Vries GJ (2011) Vasopressin innervation of the mouse (*Mus musculus*) brain and spinal cord. J Comp Neurol 519:2434–2474. 10.1002/cne.22635 21456024 PMC3939019

[B83] Rood BD, Stott RT, You S, Smith CJW, Woodbury ME, De Vries GJ (2013) Site of origin of and sex differences in the vasopressin innervation of the mouse (*Mus musculus*) brain. J Comp Neurol 521:2321–2358. 10.1002/cne.23288 23239101

[B84] Roullet FI, Wöhr M, Crawley JN (2011) Female urine-induced male mice ultrasonic vocalizations, but not scent-marking, is modulated by social experience. Behav Brain Res 216:19–28. 10.1016/j.bbr.2010.06.004 20540967 PMC3094925

[B85] Schultz RT (2005) Developmental deficits in social perception in autism: the role of the amygdala and fusiform face area. Int J Dev Neurosci 23:125–141. 10.1016/j.ijdevneu.2004.12.012 15749240

[B86] Scott N, Prigge M, Yizhar O, Kimchi T (2015) A sexually dimorphic hypothalamic circuit controls maternal care and oxytocin secretion. Nature 525:519–522. 10.1038/nature15378 26375004

[B87] Shimshek DR, Kim J, Hübner MR, Spergel DJ, Buchholz F, Casanova E, Stewart AF, Seeburg PH, Sprengel R (2002) Codon-improved Cre recombinase (iCre) expression in the mouse. Genesis 32:19–26. 10.1002/gene.10023 11835670

[B88] Södersten P, Henning M, Melin P, Ludin S (1983) Vasopressin alters female sexual behaviour by acting on the brain independently of alterations in blood pressure. Nature 301:608–610. 10.1038/301608a0 6828140

[B89] Ström JO, Theodorsson A, Ingberg E, Isaksson IM, Theodorsson E (2012) Ovariectomy and 17β-estradiol replacement in rats and mice: a visual demonstration. J Vis Exp. Advance online publication. Retrieved June 12, 2012. doi:10.3791/4013 10.3791/4013 PMC3471296 22710371

[B90] Terranova JI, Song Z, Larkin TE 2nd, Hardcastle N, Norvelle A, Riaz A, Albers HE (2016) Serotonin and arginine-vasopressin mediate sex differences in the regulation of dominance and aggression by the social brain. Proc Natl Acad Sci USA 113:13233–13238. 10.1073/pnas.1610446113 27807133 PMC5135349

[B91] Treit D, Aujla H, Menard J (1998) Does the bed nucleus of the stria terminalis mediate fear behaviors? Behav Neurosci 112:379–386. 10.1037//0735-7044.112.2.379 9588484

[B92] Unger EK, Burke KJ Jr, Yang CF, Bender KJ, Fuller PM, Shah NM (2015) Medial amygdalar aromatase neurons regulate aggression in both sexes. Cell Rep 10:453–462. 10.1016/j.celrep.2014.12.040 25620703 PMC4349580

[B93] van Leeuwen FW, Caffe AR, De Vries GJ (1985) Vasopressin cells in the bed nucleus of the stria terminalis of the rat: sex differences and the influence of androgens. Brain Res 325:391–394. 10.1016/0006-8993(85)90348-8 3978433

[B94] Van Loo PL, Mol JA, Koolhaas JM, Van Zutphen BF, Baumans V (2001) Modulation of aggression in male mice: influence of group size and cage size. Physiol Behav 72:675–683. 10.1016/s0031-9384(01)00425-5 11336999

[B110] Van Segbroeck M, Knoll AT, Levitt P, Narayanan S (2017) MUPET-Mouse Ultrasonic Profile ExTraction: A Signal Processing Tool for Rapid and Unsupervised Analysis of Ultrasonic Vocalizations. Neuron 94:465–485.e5. 10.1016/j.neuron.2017.04.005 28472651 PMC5939957

[B95] Veenema AH, Beiderbeck DI, Lukas M, Neumann ID (2010) Distinct correlations of vasopressin release within the lateral septum and the bed nucleus of the stria terminalis with the display of intermale aggression. Horm Behav 58:273–281. 10.1016/j.yhbeh.2010.03.006 20298693

[B96] Veenema AH, Bredewold R, De Vries GJ (2012) Vasopressin regulates social recognition in juvenile and adult rats of both sexes, but in sex- and age-specific ways. Horm Behav 61:50–56. 10.1016/j.yhbeh.2011.10.002 22033278 PMC3264802

[B97] Veenema AH, Bredewold R, De Vries GJ (2013) Sex-specific modulation of juvenile social play by vasopressin. Psychoneuroendocrinology 38:2554–2561. 10.1016/j.psyneuen.2013.06.002 23838102 PMC3812261

[B98] Veyrac A, Wang G, Baum MJ, Bakker J (2011) The main and accessory olfactory systems of female mice are activated differentially by dominant versus subordinate male urinary odors. Brain Res 1402:20–29. 10.1016/j.brainres.2011.05.035 21683943 PMC3155078

[B99] Wang Z, Smith W, Major DE, De Vries GJ (1994) Sex and species differences in the effects of cohabitation on vasopressin messenger RNA expression in the bed nucleus of the stria terminalis in prairie voles (*Microtus ochrogaster*) and meadow voles (*Microtus pennsylvanicus*). Brain Res 650:212–218. 10.1016/0006-8993(94)91784-1 7953686

[B100] Wei YC, Wang SR, Jiao ZL, Zhang W, Lin JK, Li XY, Li SS, Zhang X, Xu XH (2018) Medial preoptic area in mice is capable of mediating sexually dimorphic behaviors regardless of gender. Nat Commun 9:279. 10.1038/s41467-017-02648-0 29348568 PMC5773506

[B101] Wersinger SR, Kelliher KR, Zufall F, Lolait SJ, O’Carroll A-M, Young W 3rd (2004) Social motivation is reduced in vasopressin 1b receptor null mice despite normal performance in an olfactory discrimination task. Horm Behav 46:638–645. 10.1016/j.yhbeh.2004.07.004 15555506

[B102] Wersinger SR, Caldwell HK, Martinez L, Gold P, Hu S-B, Young WS 3rd (2007) Vasopressin 1a receptor knockout mice have a subtle olfactory deficit but normal aggression. Genes Brain Behav 6:540–551. 10.1111/j.1601-183X.2006.00281.x 17083331

[B103] Wilczynski W, Quispe M, Muñoz MI, Penna M (2017) Arginine vasotocin, the social neuropeptide of amphibians and reptiles. Front Endocrinol 8:186. 10.3389/fendo.2017.00186 28824546 PMC5545607

[B104] Winslow JT, Insel TR (1993) Effects of central vasopressin administration to infant rats. Eur J Pharmacol 233:101–107. 10.1016/0014-2999(93)90354-k 8472738

[B105] Wöhr M (2014) Ultrasonic vocalizations in Shank mouse models for autism spectrum disorders: detailed spectrographic analyses and developmental profiles. Neurosci Biobehav Rev 43:199–212. 10.1016/j.neubiorev.2014.03.021 24726578

[B106] Wu Z, Autry AE, Bergan JF, Watabe-Uchida M, Dulac CG (2014) Galanin neurons in the medial preoptic area govern parental behaviour. Nature 509:325–330. 10.1038/nature13307 24828191 PMC4105201

[B107] Yang CF, Chiang MC, Gray DC, Prabhakaran M, Alvarado M, Juntti SA, Unger EK, Wells JA, Shah NM (2013) Sexually dimorphic neurons in the ventromedial hypothalamus govern mating in both sexes and aggression in males. Cell 153:896–909. 10.1016/j.cell.2013.04.017 23663785 PMC3767768

